# UV-B Radiation Induces Root Bending Through the Flavonoid-Mediated Auxin Pathway in *Arabidopsis*

**DOI:** 10.3389/fpls.2018.00618

**Published:** 2018-05-17

**Authors:** Jinpeng Wan, Ping Zhang, Ruling Wang, Liangliang Sun, Wenying Wang, Huakun Zhou, Jin Xu

**Affiliations:** ^1^Key Laboratory of Tropical Plant Resources and Sustainable Use, Xishuangbanna Tropical Botanical Garden, Chinese Academy of Sciences, Mengla, China; ^2^Key Laboratory of Restoration Ecology of Cold Area in Qinghai Province, Northwest Institute of Plateau Biology, Chinese Academy of Sciences, Xining, China; ^3^University of Chinese Academy of Sciences, Beijing, China; ^4^College of Life Science, Qinghai Normal University, Xining, China

**Keywords:** ultraviolet (UV)-B, root bending, tropism, auxin, flavonoids

## Abstract

Ultraviolet (UV)-B radiation-induced root bending has been reported; however, the underlying mechanisms largely remain unclear. Here, we investigate whether and how auxin and flavonoids are involved in UV-B radiation-induced root bending in *Arabidopsis* using physiological, pharmacological, and genetic approaches. UV-B radiation modulated the direction of root growth by decreasing IAA biosynthesis and affecting auxin distribution in the root tips, where reduced auxin accumulation and asymmetric auxin distribution were observed. UV-B radiation increased the distribution of auxin on the nonradiated side of the root tips, promoting growth and causing root bending. Further analysis indicated that UV-B induced an asymmetric accumulation of flavonoids; this pathway is involved in modulating the accumulation and asymmetric distribution of auxin in root tips and the subsequent redirection of root growth by altering the distribution of auxin carriers in response to UV-B radiation. Taken together, our results indicate that UV-B radiation-induced root bending occurred through a flavonoid-mediated phototropic response to UV-B radiation.

## Introduction

Ultraviolet (UV) radiation is classified according to wavelength as UV-A radiation (320–400 nm), UV-B radiation (280–320 nm), and UV-C radiation (200–280 nm). Among these types, UV-B radiation is of prime importance because it has severely damaging effects on plant growth and development despite its small proportion (1.5% of solar radiation reaching the surface of the earth) (Nawkar et al., 2013). It is therefore essential to investigate in detail the effects of UV-B radiation on various aspects of plant growth (Solomon, 2008).

Low doses of UV-B radiation stimulate signaling through the photoreceptor *UV RESISTANCE LOCUS 8* (*UVR8*) (Rizzini et al., 2011). UVR8 interaction with CONSTITUTIVELY PHOTOMORPHOGENIC 1 (COP1) results in HY5-dependent transcriptional responses that induce the accumulation of secondary metabolites involved in protecting against UV, such as flavonoids and other phenolic compounds (Rice-Evans et al., 1997). Vandenbussche et al. (2014) found that UV-B-mediated phototropism in etiolated seedlings is regulated by both phototropin and UV-B photoreceptor UVR8, and UV-B mediates the down-regulation of the expression of auxin-responsive genes by *UVR8* pathway.

In general, roots grow in the soil to fix the plants to the ground and absorb water and nutrients (Yokawa et al., 2016). However, in nature, besides during seed germination, the roots are often exposed to sunlight because of strong wind, earthquakes, artificial factors, or animal behaviors (Yokawa et al., 2016). Red light induces a positive phototropic response, whereas blue light induces a negative phototropic response in roots. Both phototropic responses in roots, especially the red-light-induced phototropic response, are weaker than the gravitropic response and thereby frequently masked by the gravitropic response (Ruppel et al., 2001). UV-B radiation induces positive root phototropic bending (Krasylenko et al., 2012); however, the underlying physiological and molecular mechanisms remain unclear.

The asymmetrical redistribution of auxin is considered a principle regulator of the directional growth response in plants (Ruppel et al., 2001; Gilroy, 2008). The auxin influx carrier AUXIN1/LIKE AUX1 (AUX1/LAX) and the auxin efflux carrier PIN-FORMED (PIN) proteins, involved in polar auxin transport (PAT), regulate auxin distribution in the root tip, thereby determining the orientation and extent of cell division in the root meristem as well as root pattern formation (Sabatini et al., 1999). A previous study indicated that auxin plays a fundamental role in UV-disturbed morphology (Ge et al., 2010).

In addition to auxin transport, auxin perception and response also play a role in modulating the root system architecture (RSA) response to environmental cues. The auxin signal transduction pathway is activated by the binding of auxin to its receptor TRANSPORT INHIBITOR RESPONSE 1/AUXIN SIGNALING F-BOX (TIR1/AFB), promoting the degradation of Aux/IAA transcriptional repressors, releasing auxin response factors (ARFs) and activating the expression of auxin-responsive genes (Gray et al., 2001; Liu et al., 2015). Dominant mutations in several auxin/indole-3-acetic acid (Aux/IAA) genes, such as *axr2-1*, *axr3-1*, and *axr3-3* mutants, result in the inhibition of auxin signaling and disrupt root development (Gray et al., 2001; Nakamura et al., 2006). Yokawa et al. (2016) found that unilateral UV-B radiation (0.3 mW/cm^2^) induced auxin redistribution to the nonradiated side of roots. UV-B radiation affects different hormonal pathways in various ways, including biosynthesis, transport, and signaling (Vanhaelewyn et al., 2016). However, how the changes in auxin level in roots play a role in the response to UV-B stress remains to be determined.

It has been widely reported that UV-B radiation induces flavonoid production, and one of the proposed functions of flavonoids is to protect plants from potentially harmful UV irradiation (Kootstra, 1994). In fact, the UV-absorbing characteristics of flavonoids have long been considered evidence for the role of these molecules in UV protection. The purified flavonoids naringenin and rutin, as well as flavonoid extracts from apple skin, have been shown to prevent the accumulation of DNA damage (Kootstra, 1994), and plants with decreased levels of flavonoids are more sensitive to UV irradiation (Karabourniotis et al., 1992; Li et al., 1993; Winkel-Shirley, 2002).

Several studies on flavonoid mutants have also suggested a role for flavonoids in PAT (Stenlid, 1976; Jacobs and Rubery, 1988; Muday and DeLong, 2001; Biever et al., 2014). Jacobs and Rubery (1988) found that flavonoids can compete with the synthetic auxin transport inhibitor naphthylphthalamic acid (NPA) to perturb auxin transport. Yin et al. (2014) found that the flavonoid 3-*O*-rhamnoside-7-*O*-rhamnoside acts as an endogenous PAT inhibitor in *Arabidopsis* shoots. In addition, chalcone synthase (CHS)-deficient *transparent testa* (*tt*) mutants exhibit elevated auxin transport and altered growth phenotypes (Murphy et al., 2000). Santelia et al. (2008) also found that flavonoids promote asymmetric PIN shifts during gravity stimulation and thereby induce redirection of the basipetal auxin streams necessary for root bending. Silva-Navas et al. (2016) found that unilateral light induces the accumulation of flavonols to promote cell elongation and asymmetric growth in the root transition zone, suggesting that flavonols serve as positional signals. Kuhn et al. (2016) reported that *F7RhaT* (*UGT89C1*), a gene encoding a flavonol 7-*O*-rhamnosyltransferase, affects flavonol rhamnosylation and auxin metabolism, but not auxin transport. This suggests that flavonoids affect auxin distribution not only through the flavonoid-mediated auxin transport pathway but also through *F7RhaT* (*UGT89C1*)-modulated flavonol rhamnosylation and the auxin metabolism pathway. Recently, Kuhn et al. (2017) found that flavonols affect auxin transport by regulating PIN2 polarity downregulating PINOID activity.

The UV-B photoreceptor UVR8 can be expressed in roots, thereby conferring roots the ability to sense UV-B radiation (Mo et al., 2015). UV-B radiation-induced root bending toward the source of radiation has been reported in *Arabidopsis* and barley (Ktitorova et al., 2006; Krasylenko et al., 2012). However, the molecular mechanisms underlying this phenomenon remain largely unclear. The main aim of this work was to investigate the physiological and molecular responses of *Arabidopsis* roots to UV-B radiation. We found that UV-B radiation reduced auxin levels and led to an asymmetric distribution of auxin in root tips, which induced root bending. Further study indicated the involvement of flavonoids in the IAA-mediated root bending response to UV-B radiation.

## Materials and Methods

### Plant Growth and Chemical Treatments

The transgenic and mutant *Arabidopsis thaliana* lines used in this study include the following: *DR5:GFP* (Ulmasov et al., 1997), *DII-VENUS* (Brunoud et al., 2012), *HS:AXR3NT-GUS* (Gray et al., 2001), *PIN1:PIN1-GFP* (Benkova et al., 2003), *PIN2:PIN2-GFP* (Blilou et al., 2005), *PIN3:PIN3-GFP* (Žádníková et al., 2010), *PIN7:PIN7-GFP* (Blilou et al., 2005), *AUX1:AUX1-YFP* (Swarup et al., 2004), *axr3-3* (CS57505), *pin2* (CS8058), *aux1-7* (CS9583), *yucca* (Zhao et al., 2001), *uvr8-6* (Rizzini et al., 2011), and *tt4-1* (Murphy et al., 2000). The transgenic and mutant lines were confirmed using polymerase chain reaction (PCR).

*Arabidopsis* seeds were surface sterilized with 50% (v/v) bleach (containing 5% hypochlorite) for 5 min and then rinsed five times with sterile deionized water. The surface-sterilized seeds were sown onto 1/2 Murashige and Skoog (MS) agar medium [Sigma-Aldrich; supplemented with 1% (w/v) agar and 1.5% (w/v) sucrose, pH 5.75] and incubated for 3 days (d) at 4°C in the dark to synchronize germination. The seedlings were grown vertically for 5 d under standard aseptic growth conditions at 22°C with a 16 h light/8 h dark photoperiod.

Ultraviolet-B irradiation was provided by a narrowband UV-B lamps (Philips, TL20W/01-RS, 311 nm) and placed before a vertical plate with no lid and that was covered with cellulose diacetate (0.13 mm, exclude wavelengths lower than 290 nm) to completely block potential UV-C radiation (Casati and Walbot, 2008; He et al., 2011). The desired radiation was obtained by altering the distance from the plate to the lamp. The irradiance was measured by a radiometer (BNU, UV-B, China).

Five-day-old *Arabidopsis* seedlings were irradiated with 1.6 W m^-2^ UV-B for 1 h (5.76 KJ m^-2^) in the presence of simultaneous white light (photon flux density of 100 μmol m^-2^ s^-1^) with the Philips daylight lamps and then transferred to normal growth conditions for phenotypic observations. All chemicals were obtained from Sigma-Aldrich.

### GUS Staining

The beta-glucuronidase (GUS) histochemical staining was performed according to a previously described method (Hu et al., 2010). Seedlings harboring the GUS reporter gene were incubated at 37°C for 2 h in GUS staining solution with the substrate 1 mM X-Gluc (5-bromo-4-chloro-3-indolyl-β-D-GlcA cyclohexyl-ammonium). Before microscopic examination using a Zeiss Axioskop, the seedlings were incubated in 95% (v/v) ethanol to remove the chlorophyll. At least 20 seedlings were analyzed for each treatment. The experiments were repeated at least three times.

### Flavonoid Fluorescence Staining

To measure flavonoid accumulation, we incubated 5-d-old *Arabidopsis* seedlings in 2-aminoethyl diphenylborinate (DPBA) staining solution containing 0.25% (w/v) DPBA and 0.005% (v/v) Triton X-100 for 5 min as described by Murphy et al. (2000). The seedlings were then washed for 5 min with 50 mM sodium phosphate buffer [plus 0.005% (v/v) Triton X-100, pH 7.0]. After excitation with 488 nm (argon) laser, the DPBA emission was collected at 570–650 nm ranges using LSM710 (Carl Zeiss confocal fluorescence microscope) (Silva-Navas et al., 2016).

### Phenotypic Analysis

Ultraviolet-B radiation induced root bending upward from the surface of the medium, and the bending was relatively rigid. After treatment, the seedlings were carefully transferred to glass slides, and then the root tips were observed and photographed. The angle of root bending was quantified using Image J software. At least 60 replicates were measured for each treatment.

### qRT-PCR Analysis

RNA was isolated from 5-d-old frozen *Arabidopsis* seedlings using RNAiso Plus (TaKaRa) according to the manufacturer’s instructions. The concentration of RNA was quantified spectrophotometrically using a NanoQuant spectrophotometer. Reverse transcription was then performed using the PrimeScript^TM^ RT Reagent Kit with gDNA Eraser (TaKaRa). SYBR-green quantitative reverse transcription (RT)-PCR was performed with Platinum^®^ SYBR^®^ Green qPCR SuperMix-UDG (Invitrogen). *ACTIN2* (AT3G18780) and *EF1a* (AT5G60390) were used as internal controls for quantitative reverse transcription (qRT)-PCR normalization with GeNorm (Czechowski et al., 2005). The gene-specific primers are presented in Supplementary Table S1. Three independent biological replicates and three technical repetitions were performed for each gene. All primer pairs produced only one peak in the DNA melting curves, indicating high primer specificity.

### Quantification of IAA

The IAA content was quantified according to Gao et al. (2014) and Liu et al. (2015). Root tips of approximately 0.1 g fresh weight were collected and immediately frozen in liquid nitrogen. After extraction, endogenous IAAs were purified, methylated in a stream of diazomethane gas, and resuspended in 100 μL of ethyl acetate. The endogenous IAA content was analyzed using GC/MS.

### Statistical Analysis

For the fluorescence intensity analysis in the full root tips, we selected the full root tip to perform the intensity analysis. For the fluorescence intensity ratio on both sides of the root tips, we selected the left side and right side of the root tip to perform the intensity analysis, respectively, and obtained the intensity value of each side by confocal microscope. The laser power and gain values were kept the same all the time and the signals were below saturation. At least 15 roots were imaged per line for each of three repeats.

All experiments were repeated at least three times, and the results are presented as the means ± SE. The data were analyzed using Image-Pro Plus software (version 4.5.1.29; Media Cybernetics, Carlsbad, CA, United States) and SPSS (Statistic Package for Social Science) software, and the significance of differences was determined by Tukey’s test.

## Results

### UV-B Radiation Induces Positive Root Phototropic Bending

Several studies have demonstrated that UV-B-induced root bending could occur under natural situation (Ktitorova et al., 2006; Krasylenko et al., 2012). Ktitorova et al. (2006) found that unilateral UV-B radiation (1 W m^-2^ for 15 min) in barley significantly induced root bending toward the source of radiation. To determine the effects of UV-B radiation on root bending in *Arabidopsis*, 5-d-old seedlings grown vertically in 1/2 MS medium were subjected to 1–12 W m^-2^ of UV-B radiation in the presence of simultaneous white light for 15 min (0.9–10.8 KJ m^-2^). The samples were then transferred to a growth chamber maintained at 22°C under white light (photon flux density of 0.1 mmol m^-2^ s^-1^) for 4 h. We found that UV-B radiation caused the roots to bend upward from the surface of the medium and in the direction of the irradiation (Supplementary Figures S1a,b). The roots could not bend when the plate was covered with lid which excludes UV-B radiation, confirmed that root bending phenotype is resulted from UV-B radiation (data not shown).

**FIGURE 1:**
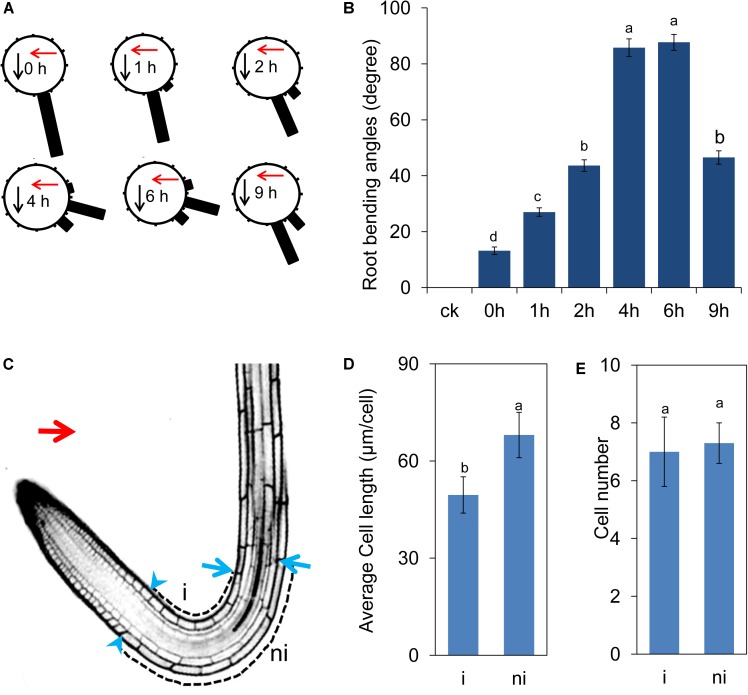
UV-B radiation induces root bending. Five-d-old *Arabidopsis* seedlings grew on vertical plates, irradiated with UV-B for 1 h and then transferred to normal growth conditions for another 9 h. **(A**,**B)** The angle of root bending was measured after 0–9 h of treatment, *n* = 60. The red arrow indicates the direction of UV-B-radiation. **(A)** Each gravistimulated root was assigned to 1 of the 12 30° sectors on a gravitropism diagram. The length of each bar represents the mean percentage of seedlings assigned to the respective sector. The red and black arrows indicate the direction of UV-B radiation and gravity, respectively. **(B)** The average root bending angles; ck, before UV-B radiation control. **(C–E)** UV-B represses cell elongation during UV-B-mediated phototropic response of *Col-0* roots **(C)**. The blue arrowheads and arrows represent the last meristematic cells and the cells where the turn initiated in the illuminated (i) and nonilluminated (ni) sides, respectively. The red arrow indicates the direction of UV-B radiation. The average cell length **(D)** and number of cells **(E)** in illuminated (i) and nonilluminated (ni) sides. The error bars represent the ±SE, and different letters indicate significantly different values (*P* < 0.01 by Tukey’s test).

Ultraviolet-B radiation induced root bending in a dosage-dependent manner (Supplementary Figure S1a). We then tested the root bending angles using 1.6 W m^-2^ UV-B radiation for 15 min–2 h (Supplementary Figure S1c). The angles of root bending peaked when we used 1.6 W m^-2^ UV-B radiation for 1 h and then transferred to a growth chamber for 4 h (the average angle was 75.56 ± 2.36) (Supplementary Figure S1c). Therefore, we selected the radiation dosage (1.6 W m^-2^ UV-B radiation for 1 h) for subsequent experiments. After the 1.6 W m^-2^ UV-B radiation for 1 h, the roots have begun to slightly bending toward the direction of UV-B radiation (0 h after 1 h-treatment) and the angles of root bending peaked at approximately 4–6 h after UV-B radiation and then gradually decreased with root elongation (**Figures 1A,B**).

To better understand the nature of root bending in response to UV-B radiation at the cellular level, we measured the length and number of cells on either side of the location of root bending. The cells on the nonradiated side were longer than those on the radiated side (**Figures 1C,D**), indicating that UV-B radiation induced a rapid response in cell elongation, thereby resulting in differential root growth on either side of the roots. However, the cell number on either side of the location of root bending was not significantly different, indicating that UV-B radiation-induced root bending did not affect cell proliferation between the two sides (**Figure 1E**).

### Auxin Is Involved in UV-B-Mediated Root Bending

**FIGURE 2:**
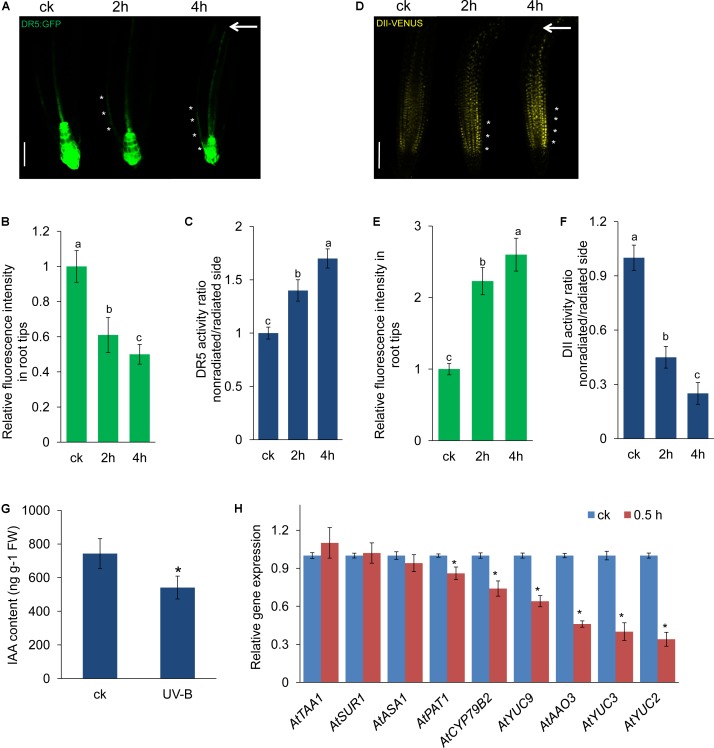
UV-B radiation affects auxin distribution in root tips. **(A–F)** GFP/YFP fluorescence in the roots of 5-d-old *DR5:GFP* **(A)** or *DII-VENUS* **(D)** seedlings exposed to UV-B radiation for 1 h and then transferred to normal growth conditions for 2–4 h, quantification of DR5:GFP **(B)** or DII-VENUS **(E)** fluorescence intensities in root tips, and ratios of DR5:GFP **(C)** or DII-VENUS **(F)** signal in the root tips on the nonradiated side versus the radiated side are shown. Bars, 60 μm. White arrows indicate the direction of UV-B radiation. White asterisks indicate more pronounced DR5:GFP signals **(A)** on the nonradiated side of roots or more pronounced DII-VENUS signals **(D)** on the radiated side; ck, before UV-B radiation control. The error bars represent the ±SE. Different letters indicate significantly different values (*P* < 0.05 by Tukey’s test). **(G)** IAA contents in the roots of wild-type seedlings exposed to UV-B radiation for 1 h and then transferred to normal growth conditions for 2 h. **(H)** Real-time quantitative reverse transcription-polymerase chain reaction (qRT-PCR) of the expression of auxin biosynthesis-related genes in the wild-type seedlings exposed to UV-B radiation for 1 h and then transferred to normal growth conditions for 0.5 h. The expression levels of the indicated genes in untreated roots (ck) were set to 1. The error bars represent the ±SE. Asterisks (^∗^) indicate significant differences with respect to the corresponding control (*P* < 0.01 by Tukey’s test).

Auxin plays a key role in modulating root growth (Petrášek and Friml, 2009; Baldwin et al., 2013), and root bending in response to UV-B radiation raised the question of whether auxin is involved in this process. Therefore, we examined auxin response using auxin-responsive *DR5:GFP* marker lines. We found a significant reduction in the expression of DR5:GFP in UV-B-treated roots (**Figures 2A,B**). Interestingly, we found that UV-B radiation also led to an asymmetric distribution of DR5:GFP fluorescence in the root tips. As shown in **Figures 2A,C**, UV-B radiation significantly increased the DR5:GFP fluorescence on the nonradiated side of roots. To confirm this finding, we also analyzed IAA perception in root tips using a transgenic line expressing the VENUS protein fused to Aux/IAA–auxin interaction domain II (*DII-VENUS*) (Brunoud et al., 2012). In the transgenic line, the VENUS signal showed a dose-dependent response to auxin (Brunoud et al., 2012), and UV-B radiation resulted in a dramatic increase in nuclear DII-VENUS fluorescence, suggesting that UV-B radiation reduced IAA perception in root tips (**Figures 2D,E**). Similarly, we found lower DII-VENUS fluorescence on the nonradiated side of the root–apex transition and elongation zones compared with the radiated side (**Figures 2D,F**). These results are consistent with Yokawa et al. (2016) and indicate that UV-B radiation led to an asymmetric auxin distribution in the root tips.

We then investigated whether UV-B radiation affects the IAA concentration in roots. As shown in **Figure 2G**, the IAA level of treated seedlings decreased by 27.2% compared with that of untreated seedlings. To investigate whether these UV-B radiation-reduced IAA concentrations were due to decreased IAA biosynthesis, we performed qRT-PCR to estimate the transcript levels of genes encoding key enzymes in the auxin biosynthesis pathway. The qRT-PCR results demonstrated that UV-B radiation decreased the transcript levels of several IAA biosynthesis genes, including *YUCCA2* (*YUC2*), *YUC3*, *YUC9*, *ABSCISIC ALDEHYDE OXIDASE3* (*AAO3*), *CYTOCHROME P450* (*CYP79B2*), and *ARABIDOPSIS HOMOLOG OF YEAST PAT1* (*PAT1*), whereas the gene expression of *TRYPTOPHAN AMINOTRANSFERASE OF ARABIDOPSIS1* (*TAA1*), *SUPERROOT 1* (*SUR1*), and *ATP SULFURYLASE ARABIDOPSIS1* (*ASA1*) was unaffected (**Figure 2H**). These data suggest that UV-B radiation resulted in down-regulation of IAA biosynthesis, which affected the auxin concentration in the roots.

**FIGURE 3:**
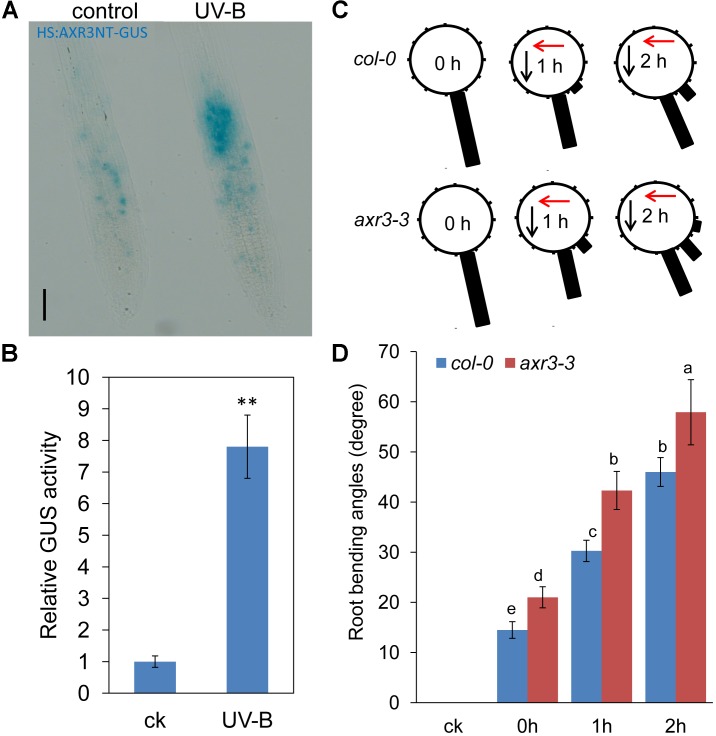
UV-B radiation increases the stabilization of Aux/IAA. **(A)** Image of GUS-staining of the roots of 5-d-old *HS:AXR3NT-GUS* seedlings. Seedlings were heat shocked at 37°C for 2 h, treated with or without UV-B radiation for 1 h, and then transferred to normal growth conditions for 45 min at 23°C, followed by GUS staining. Bars, 60 μm. **(B)** Relative GUS activity of *HS:AXR3NT-GUS*, as treated in **(A)**. The error bars represent the SE. Asterisks (^∗∗^) indicate significant differences with respect to the corresponding control (*P* < 0.01 by Tukey’s test). **(C,D)** The angle of root bending of *Col-0* and *axr3-3* seedlings irradiated with UV-B for 1 h and then transferred to normal growth conditions for 0–2 h; ck, before UV-B radiation control. *Col-0*, *n* = 60; *axr3-3*, *n* = 60. **(C)** Each gravistimulated root was assigned to 1 of the 12 30° sectors on a gravitropism diagram. The length of each bar represents the mean percentage of seedlings assigned to the respective sector. The red and black arrows indicate the direction of UV-B radiation and gravity, respectively. **(D)** The average root bending angles of *Col-0* and *axr3-3* seedlings. The error bars represent the ±SE, and different letters indicate significantly different values (*P* < 0.01 by Tukey’s test).

We next employed the *HS:AXR3-GUS* reporter line (Gray et al., 2001) to examine the effects of UV-B radiation on Aux/IAA stabilization. After heat shock, the AXR3-GUS signal was significantly increased in UV-B-treated roots (**Figures 3A,B**). These data suggest that UV-B radiation impeded auxin signaling by stabilizing Aux/IAA proteins. To further verify whether Aux/IAA proteins are involved in UV-B-induced root bending, we examined root bending in the gain-of-function *axr3-3* mutant after exposure to UV-B radiation. The *axr3-3* seedlings exhibited less suppression of PR growth after UV-B radiation than did *Col-0* seedlings, indicating that *AXR3* is involved in UV-B-induced PR growth inhibition (Supplementary Figure S2). The angles of root bending in *axr3-3* seedlings were 39.8% higher after 1 h and 25.9% higher after 2 h compared with those in the *Col-0* control seedlings subjected to UV-B radiation (**Figures 3C,D**), suggesting that the reduced auxin signaling through the increased stability of AXR3 protein increases UV-B-induced root bending.

**FIGURE 4:**
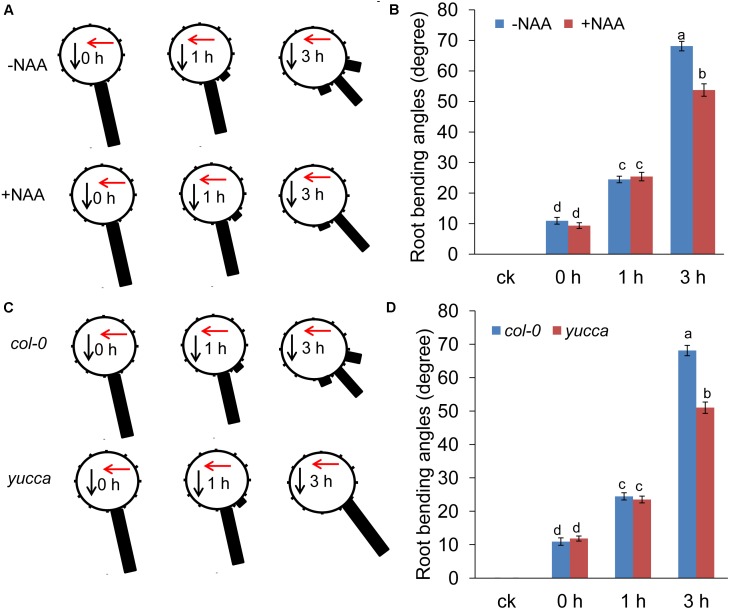
Auxin is involved in UV-B-induced root bending. **(A,B)** The angle of root bending of the wild-type *Col-0* seedlings irradiated with UV-B for 1 h and then transferred to normal growth conditions plus 0 nM NAA or 10 nM NAA for 0–3 h, *n* = 60. **(A)** Each gravistimulated root was assigned to 1 of the 12 30° sectors on a gravitropism diagram. The length of each bar represents the mean percentage of seedlings assigned to the respective sector. **(B)** The average root bending angles. **(C,D)** The angle of root bending of *Col-0* and *yucca* seedlings irradiated with UV-B for 1 h and then transferred to normal growth conditions for 0–3 h. *Col-0*, *n* = 60; *yucca*, *n* = 60. **(C)** Each gravistimulated root was assigned to 1 of the 12 30° sectors on a gravitropism diagram. The length of each bar represents the mean percentage of seedlings assigned to the respective sector. **(D)** The average root bending angles of *Col-0* and *yucca* seedlings. The red and black arrows indicate the direction of UV-B radiation and gravity, respectively; ck, before UV-B radiation control. The error bars represent the ±SE, and different letters indicate significantly different values (*P* < 0.01 by Tukey’s test).

The results presented above suggest that UV-B radiation reduced IAA accumulation in the root tips; this decreased level of auxin may be responsible for the root bending observed in the UV-B-treated seedlings. We tested this hypothesis by applying exogenous auxin. Supplementation with naphthaleneacetic acid (NAA) alleviated UV-B-induced PR growth inhibition (Supplementary Figure S3a) and alleviated UV-B-induced root bending (the average angle was 21% lower in NAA-supplemented roots compared with unsupplemented seedlings after 3 h of treatment) (**Figures 4A,B**). To confirm this finding, we also analyzed the root bending of *yucca*, an auxin over-producing mutant (Zhao et al., 2001), upon UV-B radiation. Consistent with the NAA treatment, the *yucca* mutant showed less suppression of PR growth after UV-B radiation (Supplementary Figures S3b,c) and significantly reduced root bending after exposure to UV-B radiation compared with the wild-type control (the average angles of root bending in *yucca* seedlings were 25% lower after 3 h of treatment compared with the *Col-0* control seedlings subjected to UV-B radiation) (**Figures 4C,D**).

### AUX1 and PIN2 Are Involved in Decreased Auxin Accumulation and Asymmetric Auxin Distribution in Root Tips

**FIGURE 5:**
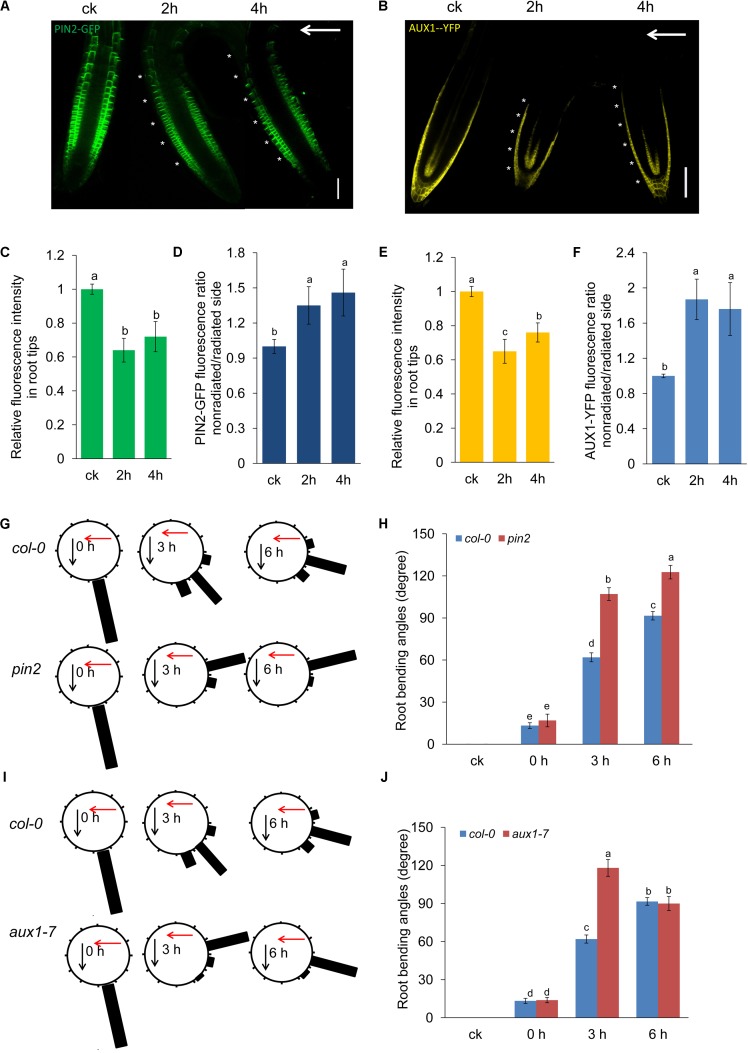
PIN2 and AUX1 are involved in UV-B-induced root bending. GFP/YFP fluorescence in the roots of 5-d-old *PIN2:GFP* **(A)** or *AUX1:YFP* **(D)** seedlings exposed to UV-B radiation for 1 h and then transferred to normal growth conditions for 2 and 4 h, quantification of PIN2:GFP **(B)** or AUX1:YFP **(E)** fluorescence intensities in root tips and ratios of PIN2:GFP **(C)** or AUX1:YFP **(F)** signals in the root tip on the nonradiated side versus the radiated side are presented. Bars, 50 μm. White arrows indicate the direction of UV-B radiation. White asterisks indicate more pronounced PIN2:GFP **(A)** or AUX1:YFP **(D)** signals on the nonradiated side of roots. **(G–I)** The angle of root bending of *Col-0*, *pin2* **(G,H)**, and *aux1-7* **(I,J)** seedlings irradiated with UV-B for 1 h and then transferred to normal growth conditions for 0–6 h. *Col-0*, *n* = 60; *pin2*, *n* = 60; *aux1-7*, *n* = 60. **(G,I)** Each gravistimulated root was assigned to 1 of the 12 30° sectors on a gravitropism diagram. The length of each bar represents the mean percentage of seedlings assigned to the respective sector. The red and black arrows indicate the direction of UV-B radiation and gravity, respectively. **(H,J)** The average root bending angles of *Col-0*, *pin2* **(H)**, and *aux1-7* **(J)** seedlings; ck, before UV-B radiation control. The error bars represent the ±SE, and different letters indicate significantly different values (*P* < 0.01 by Tukey’s test).

Mutants related to auxin transport, such as *aux1* and *pin2*, exhibit a defect in phototropic or gravitropic responses (Petrášek and Friml, 2009; Baldwin et al., 2013; Cui et al., 2013). Therefore, UV-B-induced root bending could be modulated by auxin carriers. To investigate this possibility, we examined auxin carrier levels using transgenic lines expressing *AUX1:YFP*, *PIN1:GFP*, *PIN2:GFP*, *PIN3:GFP*, and *PIN7:GFP*. We found that UV-B radiation markedly repressed the abundance of PIN2 and AUX1 (**Figures 5A,B,D,E**), whereas the abundance of PIN1, PIN3, and PIN7 was largely unaltered (Supplementary Figure S4). Furthermore, we found that signals for both PIN2:GFP and AUX1:YFP were stronger on the nonradiated side of the roots (**Figures 5A,C,D,F**).

We next investigated the roles of *PIN2* and *AUX1* in UV-B-induced root bending using *pin2* and *aux1* mutants. The loss of gravitropism in the *aux1* and *pin2* mutants led to root bending. However, different from the agravitropic root bending that cling to agar medium, UV-B radiation induced root bending upward from the surface of the medium and toward the source of radiation. Therefore, the direction of agravitropic root bending of *aux1* and *pin2* was distinct from the UV-B radiation-induced root bending. The degree of nonirradiated control (ck) root bending of *aux1* and *pin2* was 0 (it did not show any degree toward the direction of UV-B radiation). Both the *pin2* and *aux1* mutants showed less suppression of PR growth than did the *Col-0* seedlings after UV-B radiation (Supplementary Figure S5). The average angles of root bending were 72.7% higher after 3 h and 34% higher after 6 h in *pin2* seedlings (**Figures 5G,H**), and 90.5% higher after 3 h in *aux1-7* seedlings (**Figures 5I,J**) compared with the *Col-0* control seedlings subjected to UV-B radiation. These results indicate that PIN2 and AUX1 are involved in generating the asymmetric auxin distribution underlying the root bending response to UV-B radiation.

To further confirm the effect of auxin transport in root bending, we also used NPA, an auxin transport inhibitor. The average angles of root bending of NPA-treated *Col-0* seedlings were 16.8% higher after 4 h and 28.6% higher after 6 h compared with the control (NPA-untreated) seedlings subjected to UV-B radiation (Supplementary Figure S6).

### Involvement of Flavonoids in UV-B-Disturbed Auxin Distribution in Root Tips

To further investigate the molecular mechanisms underlying UV-B-induced root bending, we analyzed the transcript profiles in roots via high-throughput RNA-seq (Supplementary Materials and Methods) followed by qRT-PCR. We compared the transcripts obtained at 0.5 and 2 h after UV-B treatment. Relative to the gene expression levels under control conditions, 1436 genes were down-regulated and 557 genes were up-regulated in roots after 0.5 h of UV-B treatment; 1710 genes were down-regulated, and 1040 genes were up-regulated in roots after 2 h of UV-B treatment (Supplementary Figure S7 and Supplementary Table S2). The differentially expressed genes showed enrichment in the Kyoto Encyclopedia of Genes and Genomes (KEGG) pathways of photosynthesis, carbon fixation, and flavonoid biosynthesis, among others, due to either 0.5 or 2 h of UV-B treatment (Supplementary Figure S8 and Supplementary Table S3). The qRT-PCR results strongly agreed with the RNA-seq results (*R*^2^ = 0.6339); this finding verified the accuracy of the RNA-seq results (Supplementary Figure S9).

**FIGURE 6:**
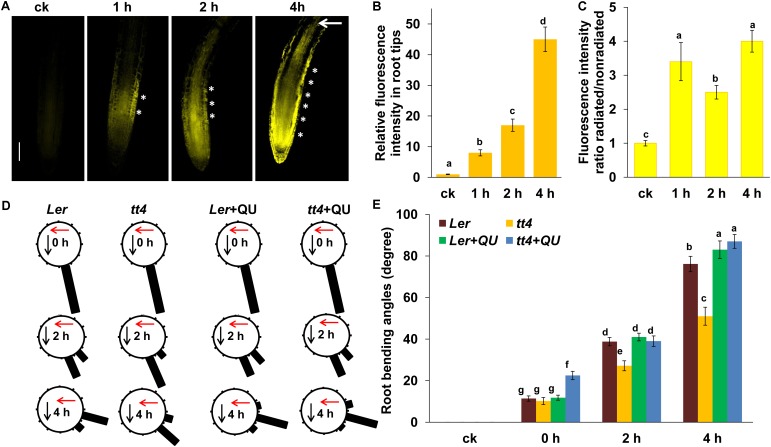
Flavonoids are involved in UV-B-induced root bending. **(A)** DPBA fluorescence in the roots of 5-d-old wild-type *Col-0* seedlings exposed to UV-B radiation for 1 h and then transferred to normal growth conditions for 1, 2, and 4 h. Bars, 100 μm. **(B,C)** Quantification of DPBA fluorescence intensities in root tips **(B)**, and ratios of DPBA fluorescence intensities in the root tips on the radiated side versus the nonradiated side **(C)** are shown. **(D,E)** The angle of root bending of *Ler* and *tt4-1* seedlings irradiated with UV-B for 1 h and then transferred to normal growth conditions plus 0 nM QU or 100 nM QU for 1–4 h, *n* = 60. **(D)** Each gravistimulated root was assigned to 1 of the 12 30° sectors on a gravitropism diagram. The length of each bar represents the mean percentage of seedlings assigned to the respective sector. The red and black arrows indicate the direction of UV-B radiation and gravity, respectively. **(E)** The average root bending angles of *Ler* and *tt4-1* seedlings; ck, before UV-B radiation control. White asterisks indicate more pronounced DPBA fluorescence on the radiated side of roots. The error bars represent the ±SE, and different letters indicate significantly different values (*P* < 0.01 by Tukey’s test).

The RNA-seq analysis showed that UV-B radiation significantly induced the expression of flavonoid biosynthesis-related genes, and the results were consistent with previous reports that UV-B activates the expression of flavonoid biosynthesis-related genes (Rice-Evans et al., 1997; Nawkar et al., 2013). As it has been documented that flavonoids can affect auxin distribution (Jacobs and Rubery, 1988; Muday and DeLong, 2001) and root phototropism (Silva-Navas et al., 2016), the possible role of flavonoids in UV-B-induced root bending was also investigated in our study. First, flavonoids were stained with diphenylborinic acid 2-aminoethyl ester (DPBA), which predominantly detects quercetin (QU) and kaempferol, two natural flavonoids in plants (Murphy et al., 2000; Lewis et al., 2011). DPBA fluorescence was dramatically increased in the roots of *Col-0* seedlings after UV-B radiation compared with the untreated controls (**Figure 6**). Interestingly, we found that UV-B radiation led to an asymmetric distribution of flavonoid in the root tips. As shown in **Figure 6**, UV-B radiation significantly increased DPBA fluorescence on the radiated side of roots.

To investigate whether flavonoids are involved in UV-B-induced root bending, we analyzed root bending in the flavonoid biosynthesis-defective mutant *transparent testa 4-1*(*tt4-1*) after UV-B radiation. The *tt4-1* mutant exhibited significantly lower flavonoid levels in the roots compared with wild-type seedlings following exposure to UV-B radiation, as indicated by DPBA fluorescence (Supplementary Figure S10). The *tt4-1* mutant showed markedly greater suppression of PR growth than did the wild-type seedlings after UV-B radiation (Supplementary Figure S11). The *tt4-1* mutant also showed significantly reduced root bending after UV-B radiation compared with the wild-type control. The average angles of root bending were 30% lower after 2 h, and 33% lower after 4 h in *tt4-1* seedlings compared with the wild-type control seedlings subjected to UV-B radiation (**Figures 6B,C**).

To further confirm this observation, we also analyzed the effect of exogenous QU, a natural flavonoid, on UV-B radiation-induced root bending. Exogenous application of QU resulted in a greater degree of root bending compared with UV-B radiation alone (the average angles of root bending were 6 and 43.6% higher after 2 h, and 9 and 71% higher after 4 h in QU-supplemented *Ler* and *tt4* seedlings, respectively, compared with unsupplemented seedlings subjected to UV-B radiation), and the QU-supplemented *tt4* mutant showed a similar root bending degree compared with the QU-supplemented *Ler* seedlings after 2 h of UV-B radiation (**Figures 6B,C**). These data indicate that UV-B-induced root bending occurs, at least partially, through the UV-B-mediated rapid accumulation of flavonoids in roots.

**FIGURE 7:**
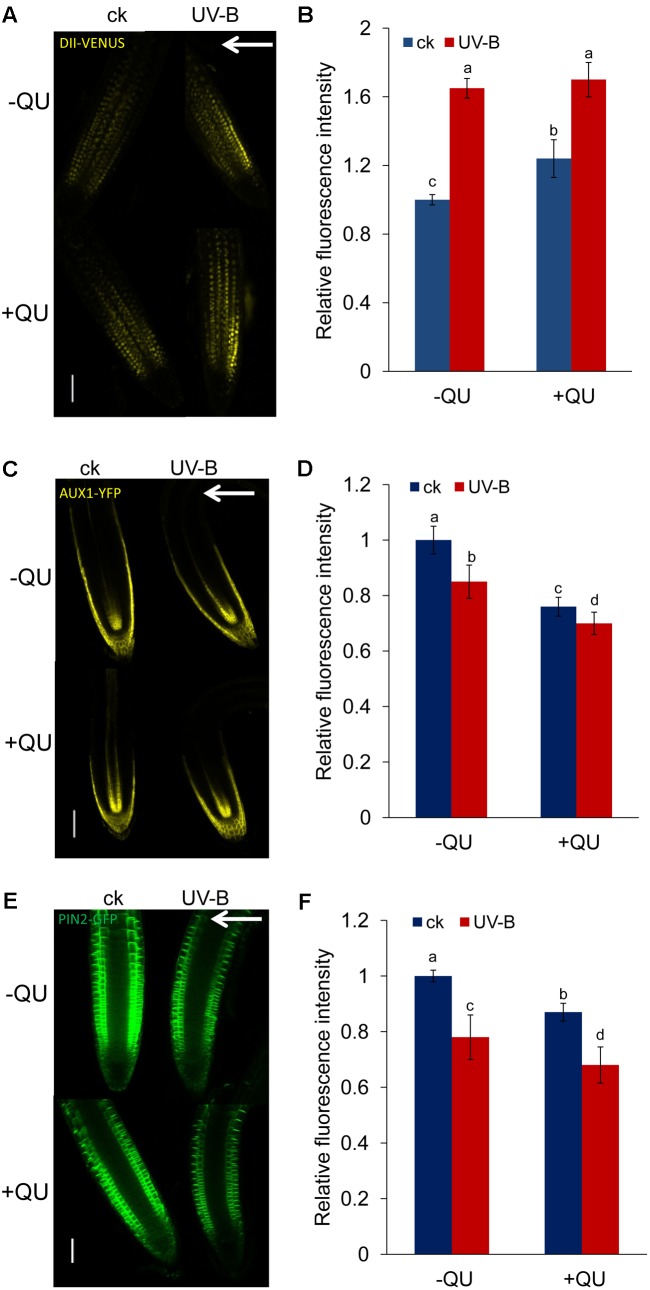
UV-B radiation influences auxin accumulation and transport via flavonoids. GFP/YFP fluorescence in the roots of 5-d-old *DII-VENUS* **(A)**, *AUX1:YFP* **(C)**, and *PIN2:GFP* **(E)** seedlings exposed to 1.6 W m^-2^ UV-B radiation for 1 h and then transferred to normal growth conditions plus 0 nM QU or 100 nM QU for 4 h. Quantification of DII-VENUS **(B)**, AUX1:YFP **(D)**, and PIN2:GFP **(F)** fluorescence intensities in the root tips is presented. Bars, 50 μm. White arrows indicate the direction of UV-B-radiation; ck, before UV-B radiation control. The error bars represent the SE, and different letters indicate significantly different values (*P* < 0.05 by Tukey’s test).

We next examined whether and how flavonoids modulate auxin distribution in root tips subjected to UV-B radiation. We first used an auxin-perceptive *DII-VENUS* marker line to monitor possible changes in auxin distribution in UV-B-treated roots in the presence or absence of exogenous QU. UV-B radiation reduced auxin distribution in root tips, and treatment with QU alone increased the *DII-VENUS* expression in root tips, indicating that exogenous QU reduces the distribution of auxin in root tips (**Figures 7A,B**). Nonetheless, supplementation with QU did not significantly increase DII-VENUS expression in UV-B-treated roots. It might be that UV-B induces dramatic accumulation of flavonoids in root tips; thus, exogenous flavonoid cannot further impact the phenotype.

The results above suggest that the UV-B-regulated auxin distribution involved in root bending is modulated by *AUX1* and *PIN2*. To further explore the role of QU in the auxin distribution response to UV-B radiation, we analyzed the levels of AUX1 and PIN2 expression in UV-B-treated roots in the presence or absence of exogenous QU using transgenic lines expressing *AUX1:YFP* and *PIN2:GFP*. Treatment with QU alone markedly decreased AUX1:YFP (**Figures 7C,D**) and PIN2:GFP (**Figures 7E,F**) fluorescence, and QU supplementation further reduced AUX1 and PIN2 level in UV-B-treated roots (**Figures 7C–F**), suggesting that QU reduced auxin transport by affecting the levels of these auxin carriers.

### *UVR8* Is Involved in UV-B-Mediated Root Bending

**FIGURE 8:**
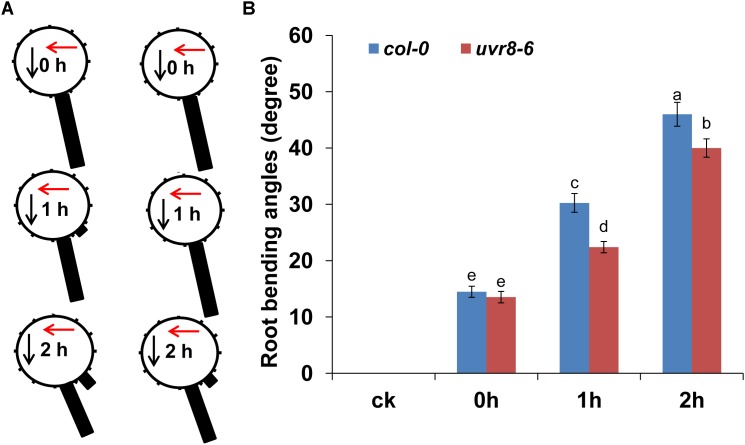
*UVR8* is involved in UV-B-induced root bending. The angle of root bending of *col-0* and *uvr8-6* mutant seedlings irradiated with UV-B for 1 h and then transferred to normal growth conditions for 0–2 h. *col-0*, *n* = 60; *uvr8-6*, *n* = 60. **(A)** Each gravistimulated root was assigned to 1 of the 12 30° sectors on a gravitropism diagram. The length of each bar represents the mean percentage of seedlings assigned to the respective sector. The red and black arrows indicate the direction of UV-B-radiation and gravity, respectively. **(B)** The average root bending angles of *col-0* and *uvr8-6* seedlings; ck, before UV-B radiation control. The error bars represent the ±SE, and different letters indicate significantly different values (*P* < 0.01 by Tukey’s test).

Ultraviolet-B radiation activates *MPK3* and *MPK6* via the *MKP1* signaling pathway (González Besteiro et al., 2011; Nawkar et al., 2013). Exposure to UV-B also initiates signaling through the *UVR8* pathway (Rizzini et al., 2011). The stress-induced MAPK pathway and the UVR8-mediated photomorphogenesis pathway are independent of each other and coordinately determine plant UV-B tolerance. Thus, we used *mkp1*, *mpk3*, *mpk6*, and *uvr8-6* mutants to ascertain whether UV-B-mediated root bending is *MKP1* dependent or *UVR8* dependent. Although the root bending response to UV-B radiation was similar in *mkp1*, *mpk3*, and *mpk6* mutants compared with the wild-type plants (Supplementary Figure S12), a lower response was observed in *uvr8-6* (**Figure 8**). The average angles of root bending in *uvr8-6* seedlings were 30% lower after 1 h and 13% lower after 2 h compared with the *col-0* control seedlings subjected to UV-B radiation. Because the *uvr8* mutant is hypersensitive to UV-B radiation, we analyzed whether UV-B radiation induced root growth cessation. After UV-B radiation, both *uvr8-6* and *col-0* seedlings showed a reduced primary root (PR) growth, and the PR growth of *uvr8-6* seedlings gradually recovered to a similar level compared with *col-0* seedling after 4 d of treatment when the UV-B-radiated seedlings were transferred to normal condition (Supplementary Figure S13), indicating that UV-B radiation did not result in root growth cessation in the *uvr8* mutant. Taken together, these data indicate that UV-B-mediated root bending is at least partially dependent on the *UVR8* signaling pathway.

Previous studies have reported that UV-B activates the expression of flavonoid biosynthesis-related genes through the *UVR8* signaling pathway (Rice-Evans et al., 1997; Nawkar et al., 2013). We thus also analyzed flavonoid accumulation in *uvr8* roots. As shown in Supplementary Figure S10, the DPBA fluorescence was significantly lower in the roots of the *uvr8-6* mutant compared with the wild-type seedlings after UV-B radiation, indicating that UV-B-induced flavonoid production depends on *UVR8*.

## Discussion

Stress-induced root bending is a common phenomenon in plants (Li and Zhang, 2008). Salt modulates root growth direction by inducing root bending as a salt-avoidance tropism (Li and Zhang, 2008). Light locally induces a root light avoidance mechanism, allowing roots to bend and escape from the light (Zhang et al., 2013; Silva-Navas et al., 2016). Different from the avoidance tropism of root bending, UV-B induces root bending toward the irradiated direction. It is well known that the penetration capacity of UV radiation is limited. Brumfield (1953) found that the cell division of only the outer layer of the root tip meristem was suppressed when roots were irradiated with UV-C because these cells were not protected by the root cap. Ktitorova et al. (2006) also found that UV-B radiation-induced cell division cessation and cell vacuolation did not occur in the distal meristem zone, which is protected by the root cap. Therefore, in UV-B radiation-induced root bending toward the irradiated direction, the root cap would increase the protection of root meristem cells from UV-B radiation.

### UV-B Radiation Induces Root Bending by Modulating Auxin Perception and Distribution in Roots

Auxin perception and signaling both play roles in tropistic responses (Band et al., 2012). Disrupting the auxin responsiveness of expanding epidermal cells by expressing a mutant form of the Aux/IAA17 protein, the *axr3-1* mutant lacks root gravitropism (Swarup et al., 2005). Vandenbussche et al. (2014) found that UV-B radiation results in the down-regulation of the expression of auxin-responsive genes. Our results indicate that UV-B perturbs auxin signaling by stabilizing Aux/IAA proteins, as indicated by HS:AXR3-GUS expression, and the gain-of-function *axr3-3* mutant showed a greater extent of root bending than did the wild-type control. These results support the hypothesis that UV-B radiation induces root bending by reducing auxin signaling through increase of Aux/IAA stabilization in roots. Further study will investigate whether other Aux/IAA proteins are also UV-B target and involved in UV-B-mediated auxin signaling.

In addition to auxin perception, the auxin content in roots and the asymmetric distribution of auxin are known to contribute to root bending during tropistic responses (Sun et al., 2008). These effects are potentially regulated by the expression of auxin biosynthesis-related genes and auxin carriers (Abas et al., 2006). Indeed, UV-B radiation decreased the transcript levels of several IAA biosynthesis genes, such as *YUC2*, *YUC3*, *YUC9*, *AAO3*, *CYP79B2*, and *PAT1*, suggesting that UV-B radiation decreases auxin content in roots by down-regulating IAA biosynthesis-related gene expression. Mutants related to auxin transport, such as *aux1* and *pin2*, exhibit defects in gravitropic responses (Petrášek and Friml, 2009; Haga and Sakai, 2012). Previous studies showed that an asymmetric distribution of auxin carriers is needed to generate asymmetric auxin distribution during root gravitropism and the root phototropic response (Zhang et al., 2013). In this study, we observed reduced auxin accumulation in root tips and asymmetric auxin distribution during the root bending response to UV-B radiation. We found that UV-B radiation increased auxin distribution on the nonradiated side of the root–apex transition and elongation zones; at this site, auxin promoted growth and caused root bending by asymmetric cell elongation. Further examination showed that UV-B radiation induced the asymmetric distribution of AUX1 and PIN2 on both sides of the root tips. The nonradiated side of the roots showed stronger PIN2:GFP and AUX1:YFP signals than did the radiated side, suggesting a role for these increased PIN2 and AUX1 levels in the higher auxin distribution observed on the nonradiated side. Our findings demonstrate that a reduction in the abundance and asymmetric distribution of AUX1 and PIN2 in roots exposed to UV-B radiation may interfere with the distribution of auxin within root meristem cells as well as auxin transport during the root bending response to UV-B radiation. Such changes may promote root bending to modify the direction of root growth. Mutation of *AUX1* and *PIN2* would disrupt auxin transport and reduce the asymmetric auxin distribution in UV-B-radiated roots. However, we found that *aux1-7* and *pin2* mutants are more sensitive to UV-B-induced root bending than *Col-0* plants. These results indicated that, in addition to asymmetric distribution of auxin induced by UV-B, reduction of auxin accumulation in UV-B-radiated root tips also led to root agravitropic response, and thereby aggravating root bending. However, the detailed molecular mechanisms involved in how the modulation of the root agravitropic response are involved in UV-B-mediated root bending and the possible interaction between root agravitropic response and phototropic bending in response to UV-B radiation remain to be further explored.

Despite the asymmetric auxin distribution during root gravitropism and the root phototropic response-induced root bending, auxin accumulated on the concave side of gravistimulated roots, but it accumulated on the convex side of the roots in response to unilateral blue light stimulation (Zhang et al., 2013) and UV-B radiation (in this study). The difference between the gravitropism assays and UV-B radiation may be due to the following reasons: (1) UV-B radiation significantly reduced auxin accumulation in roots by repressing auxin biosynthesis (evidence from qRT-PCR), transport (evidence from AUX1:YFP and PIN2-GFP), and signaling (evidence from *HS:AXR3-GUS* reporter), and thereby inhibited root growth. (2) Unilateral UV-B radiation significantly increased auxin distribution on the nonradiated side (convex side) of the roots. Greater auxin accumulation on the nonradiated side (convex side) of roots resulted in higher H^+^ efflux, thereby promoting cell wall acidification on the side (Rubery and Sheldrake, 1974; Yan et al., 2016) and ultimately leading to asymmetric growth and subsequent root bending. Taken together, these data indicate that although UV-B radiation reduces total auxin accumulation in roots, it increases auxin distribution on the nonradiated side (convex side) of roots, thereby promoting growth on the nonradiated side (convex side) and ultimately resulting in root growth toward the radiation.

### Asymmetric Flavonoid Accumulation in Roots Is Associated With UV-B-Mediated Root Bending Through Decreased Auxin Accumulation and Induction of Asymmetric Auxin Distribution in Root Tips

It is believed that one of the important aspects by which flavonoids protect plants from UV-B irradiation is their UV-absorbing characteristics (Kootstra, 1994). Flavonoids can be synthesized in the root elongation zone and accumulate in the root tips of plants subjected to UV-B radiation (Karabourniotis et al., 1992; Li et al., 1993; Winkel-Shirley, 2002). Santelia et al. (2008) found that flavonoids could induce an asymmetric distribution of auxin in root tips, thus resulting in root bending. UV-B radiation induces positive root phototropic bending. A possible explain is that UV-B causes a destruction of auxin on the illuminated side of the roots, and UV-B would likely not penetrate to the shaded side of the tissue and thus an asymmetric IAA distribution would result. UV-B radiation markedly induces flavonoid production and the increased flavonoids would protect roots from UV-B irradiation. Therefore, we wondered whether the flavonoids play a protective role in UV-B-irradiated roots, or flavonoid itself also plays a role in modulating the root system development response to UV-B radiation. Indeed, we found that UV-B-induced flavonoids affected auxin distribution by altering the abundance of auxin carriers in the root tips, thereby modulating the direction of root growth. Several lines of evidence support this conclusion. First, we demonstrated that flavonoids are needed for UV-B-induced root bending. The flavonoid biosynthesis-defective mutant *tt4* showed significantly reduced root bending in response to UV-B radiation compared with the wild-type controls. A similar result was also reported by Silva-Navas et al. (2016), i.e., that the *tt4* mutant showed a reduced root phototropic response to light. Second, physiological analysis showed that exogenous application of QU reduced auxin accumulation in the root tips and resulted in a greater extent of root bending compared with UV-B radiation alone. Third, exogenous QU supplementation reduced the abundance of AUX1 and PIN2 in root tips. Fourth, we confirmed that UV-B-induced flavonoid production in the root tips depends on *UVR8*, and loss of function *uvr8* mutant shows a reduced response to UV-B radiation. Silva-Navas et al. (2016) found that light induced an asymmetric accumulation of flavonoids, thereby resulting in asymmetric growth in the root transition zone. Consistent with their results, we also observed an asymmetric accumulation of flavonoids in the UV-B-radiated root tips. UV-B radiation significantly increased flavonoid production on the radiated side of roots, as indicated by DPBA fluorescence. The increased accumulation of flavonoids on the radiated side of roots resulted in reduced auxin transport and subsequently reduced auxin distribution on that side, and ultimately asymmetric root growth.

Yokawa et al. (2016) found that UV-B radiation induced ROS accumulation in the root tips. PR growth could be regulated by the auxin pathway and ROS pathway independently (Tsukagoshi et al., 2010). In addition to acting as auxin transport inhibitors, flavonoids could also act as ROS scavengers. ROS could also induce flavonoid production (Silva-Navas et al., 2016). Further study will elucidate the possible interaction between ROS and flavonoid in mediating UV-B-induced root bending.

We found that the *uvr8* mutant displayed less root bending compared with the wild-type control under UV-B radiation. Consistent with these results, the *uvr8* mutant accumulated a lower level of flavonoids in the roots compared with the wild-type control in response to UV-B radiation. A previous study reported that the single mutants *mpk3* and *mpk6* exhibited enhanced UV-B tolerance, suggesting a genetically defined *in vivo* role for these kinases in UV-B stress signaling (González Besteiro et al., 2011). We found that the *mkp1*, *mpk3*, and *mpk6* single mutants exhibited a root bending phenotype that was similar to that of the wild-type control. These results suggest that UV-B-induced root bending depends on the *UVR8* signaling pathway, but not on the *MKP1*–*MPK3/6* signaling pathway.

In this study, we have showed that UV-B radiation inhibits PR growth and induces root bending. We found that most of mutants that showed a higher root bending also had a less inhibition of PR growth exposed to UV-B radiation. These results support the hypothesis that the root cap would increase the protection of root meristem cells from UV-B radiation when the root bending toward the irradiated direction, and thereby alleviating UV-B-induced PR growth inhibition. Another possible reason is that the more rapid growth rate would result in higher root bending. However, we found that the auxin over-producing *yucca* mutant that had a lower root bending but it showed a decrease in PR growth inhibition. These results support the view that the root growth and bending in response to gravity, light, or abiotic stresses occurs through multiple overlapping mechanisms and that UV-B radiation may act as an input for one of multiple responses (Wolverton et al., 2002; Bai et al., 2013).

**FIGURE 9:**
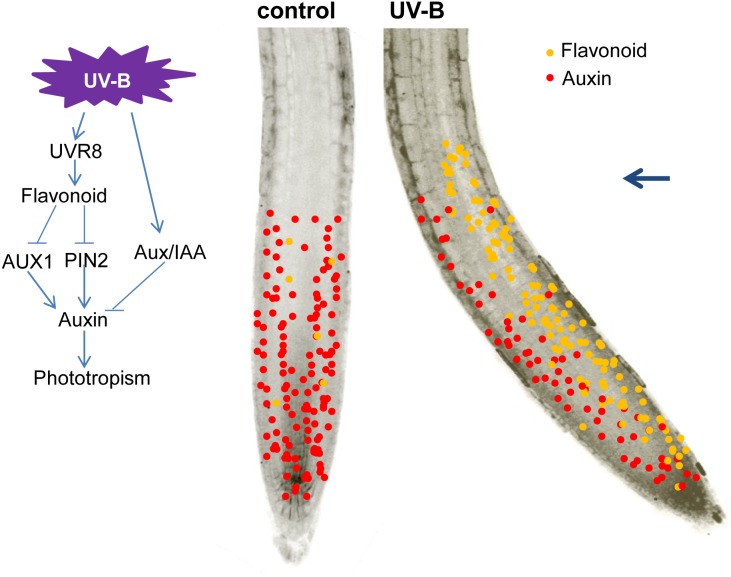
A proposed model of UV-B-mediated root bending. Blue arrow indicates the direction of UV-B radiation.

In summary, our data indicate that *UVR8*-dependent flavonoid production and its asymmetric accumulation in root tips are associated with UV-B-mediated root phototropic bending through decreased auxin accumulation and induction of asymmetric auxin distribution in root tips by modulating the distribution of AUX1 and PIN2 (**Figure 9**). These findings provide new insight into how UV-B radiation regulates root growth through a flavonoid-mediated phototropic response to UV-B radiation.

## Author Contributions

JX conceived the study and designed the experiments. JW, PZ, RW, and LS carried out the experiments. PZ, JW, RW, HZ, WW, and JX analyzed the data. JX wrote the manuscript. PZ, JW, HZ, WW, and JX revised the manuscript.

## Conflict of Interest Statement

The authors declare that the research was conducted in the absence of any commercial or financial relationships that could be construed as a potential conflict of interest.

## References

[B1] Abas L. Benjamins R. Malenica N. Paciorek T. Wirniewska J. Moulinier-Anzola J. C. (2006). Intracellular trafficking and proteolysis of the *Arabidopsis* auxin-efflux facilitator PIN2 are involved in root gravitropism. 8 249–256. 10.1038/ncb1369 16489343

[B2] Bai H. Murali B. Barber K. Wolverton C. (2013). Low phosphate alters lateral root setpoint angle and gravitropism. 100 175–182. 10.3732/ajb.1200285 23125433

[B3] Baldwin K. L. Strohm A. K. Masson P. H. (2013). Gravity sensing and signal transduction in vascular plant primary roots. 100 126–142. 10.3732/ajb.1200318 23048015

[B4] Band L. R. Wells D. M. Larrieu A. Sun J. Middleton A. M. French A. P. (2012). Root gravitropism is regulated by a transient lateral auxin gradient controlled by a tipping-point mechanism. 109 4668–4673. 10.1073/pnas.1201498109 22393022 PMC3311388

[B5] Benkova E. Michniewicz M. Sauer M. Teichmann T. Seifertova D. Jurgens G. (2003). Local, efflux-dependent auxin gradients as a common module for plant organ formation. 115 591–602. 10.1016/S0092-8674(03)00924-3 14651850

[B6] Biever J. J. Brinkman D. Gardner G. (2014). UV-B inhibition of hypocotyl growth in etiolated *Arabidopsis thaliana* seedlings is a consequence of cell cycle arrest initiated by photodimer accumulation. 65 2949–2961. 10.1093/jxb/eru035 24591052 PMC4056539

[B7] Blilou I. Xu J. Wildwater M. Willemsen V. Paponov I. Friml J. (2005). The PIN auxin efflux facilitator network controls growth and patterning in *Arabidopsis* roots. 433 39–44. 10.1038/nature03184 15635403

[B8] Brumfield R. T. (1953). The effect of ultraviolet irradiation on cell division and elongation in timothy roots. 39 366–370. 10.1073/pnas.39.5.366 16589278 PMC1063792

[B9] Brunoud G. Wells D. M. Oliva M. Larrieu A. Mirabet V. Burrow A. H. (2012). A novel sensor to map auxin response and distribution at high spatio-temporal resolution. 482 103–106. 10.1038/nature10791 22246322

[B10] Casati P. Walbot V. (2008). Maize lines expressing RNAi to chromatin remodeling factors are similarly hypersensitive to UV-B radiation but exhibit distinct transcriptome responses. 3 216–229. 10.4161/epi.3.4.6631 18719398 PMC2551322

[B11] Cui D. Zhao J. Jing Y. Fan M. Liu J. Wang Z. (2013). The Arabidopsis IDD14, IDD15, and IDD16 cooperatively regulate lateral organ morphogenesis and gravitropism by promoting auxin biosynthesis and transport. 9:e1003759. 10.1371/journal.pgen.1003759 24039602 PMC3764202

[B12] Czechowski T. Stitt M. Altmann T. Udvardi M. K. Scheible W. R. (2005). Genome-wide identification and testing of superior reference genes for transcript normalization in Arabidopsis. 139 5–17. 10.1104/pp.105.063743 16166256 PMC1203353

[B13] Gao X. Yuan H. M. Hu Y. Q. Li J. Lu Y. T. (2014). Mutation of *Arabidopsis CATALASE2* results in hyponastic leaves by changes of auxin levels. 37 175–188. 10.1111/pce.12144 23738953

[B14] Ge L. Peer W. Robert S. Swarup R. Ye S. Prigge M. (2010). Arabidopsis *ROOT UVB SENSITIVE2/WEAK AUXIN RESPONSE1* is required for polar auxin transport. 22 1749–1761. 10.1105/tpc.110.074195 20562234 PMC2910957

[B15] Gilroy S. (2008). Plant tropisms. 18 R275–R277. 10.1016/j.cub.2008.02.033 18397730

[B16] González Besteiro M. A. Bartels S. Albert A. Ulm R. (2011). Arabidopsis MAP kinase phosphatase 1 and its target MAP kinases 3 and 6 antagonistically determine UV-B stress tolerance, independent of the UVR8 photoreceptor pathway. 68 727–737. 10.1111/j.1365-313X.2011.04725.x 21790814

[B17] Gray W. M. Kepinski S. Rouse D. Leyser O. Estelle M. (2001). Auxin regulates SCF^TIR1^-dependent degradation of AUX/IAA proteins. 414 271–276. 10.1038/35104500 11713520

[B18] Haga K. Sakai T. (2012). PIN auxin efflux carriers are necessary for pulse-induced but not continuous light-induced phototropism in Arabidopsis. 160 763–776. 10.1104/pp.112.202432 22843667 PMC3461554

[B19] He J. M. Zhang Z. Wang R. B. Chen Y. P. (2011). UV-B-induced stomatal closure occurs via ethylene-dependent NO generation in *Vicia faba*. 38 293–302. 10.1071/FP10219 32480885

[B20] Hu Y. Q. Liu S. Yuan H. M. Li J. Yan D. W. Zhan J. F. (2010). Functional comparison of catalase genes in the elimination of photorespiratory H_2_O_2_ using promoter- and 3′- untranslated region exchange experiments in the *Arabidopsis cat2* photorespiratory mutant. 33 1656–1670. 10.1111/j.1365-3040.2010.02171.x 20492555

[B21] Jacobs M. Rubery P. H. (1988). Naturally occurring auxin transport regulators. 241 346–349. 10.1126/science.241.4863.346 17734864

[B22] Karabourniotis G. Papadopoulos K. Papamarkou M. Manetas Y. (1992). Ultraviolet-B radiation absorbing capacity of leaf hairs. 86 414–418. 10.1111/j.1399-3054.1992.tb01337.x

[B23] Kootstra A. (1994). Protection from UV-B-induced DNA damage by flavonoids. 26 771–774. 10.1007/BF00013762 7948931

[B24] Krasylenko Y. A. Yemets A. I. Sheremet Y. A. Blume Y. B. (2012). Nitric oxide as a critical factor for perception of UV-B irradiation by microtubules in *Arabidopsis*. 145 505–515. 10.1111/j.1399-3054.2011.01530.x 21973209

[B25] Ktitorova I. N. Demchenko N. P. Kalimova I. B. Demchenko K. N. Skobeleva O. V. (2006). Cellular analysis of UV-B-induced barley root subapical swelling. 53 824–836. 10.1134/S1021443706060148

[B26] Kuhn B. M. Errafi S. Bucher R. Dobrev P. Geisler M. Bigle R. L. (2016). 7-Rhamnosylated Flavonols modulate homeostasis of the plant hormone auxin and affect plant development. 291 5385–5395. 10.1074/jbc.M115.701565 26742840 PMC4777868

[B27] Kuhn B. M. Nodzyński T. Errafi S. Bucher R. Gupta S. Ringli C. (2017). Flavonol-induced changes in PIN2 polarity and auxin transport in the *Arabidopsis thaliana* rol1-2 mutant require phosphatase activity. 7:41906. 10.1038/srep41906 28165500 PMC5292950

[B28] Lewis D. R. Ramirez M. V. Miller N. D. Vallabhaneni P. Ray W. K. Helm R. F. (2011). Auxin and ethylene induce flavonol accumulation through distinct transcriptional networks. 156 144–164. 10.1104/pp.111.172502 21427279 PMC3091047

[B29] Li J. Ou-Lee T. M. Raba R. Amundson R. G. Last R. L. (1993). Arabidopsis flavonoid mutants are hypersensitive to UV-B irradiation. 5 171–179. 10.1105/tpc.5.2.171 12271060 PMC160260

[B30] Li X. Zhang W. S. (2008). Salt-avoidance tropism in *Arabidopsis thaliana*. 3 351–353. 10.4161/psb.3.5.5371 19841669 PMC2634281

[B31] Liu W. Li R. J. Han T. T. Cai W. Fu Z. W. Lu Y. T. (2015). Salt stress reduces root meristem size by nitric oxide-mediated modulation of auxin accumulation and signaling in Arabidopsis. 168 343–356. 10.1104/pp.15.00030 25818700 PMC4424022

[B32] Mo M. Yokawa K. Wan Y. Baluška F. (2015). How and why do root apices sense light under the soil surface? 6:775. 10.3389/fpls.2015.00775 26442084 PMC4585147

[B33] Muday G. K. DeLong A. (2001). Polar auxin transport: controlling where and how much. 6 535–542. 10.1016/S1360-1385(01)02101-X 11701382

[B34] Murphy A. Peer W. A. Taiz L. (2000). Regulation of auxin transport by aminopeptidases and endogenous flavonoids. 211 315–324. 10.1007/s004250000 10987549

[B35] Nakamura A. Nakajima N. Goda H. Shimada Y. Hayashi K. Nozaki H. (2006). Arabidopsis *Aux/IAA* genes are involved in brassinosteroid-mediated growth responses in a manner dependent on organ type. 45 193–205. 10.1111/j.1365-313X.2005.02582.x 16367964

[B36] Nawkar G. M. Maibam P. Park J. H. Sahi V. P. Lee S. Y. Kang C. H. (2013). UV-induced cell death in plants. 14 1608–1628. 10.3390/ijms14011608 23344059 PMC3565337

[B37] Petrášek J. Friml J. (2009). Auxin transport routes in plant development. 136 2675–2688. 10.1242/dev.030353 19633168

[B38] Rice-Evans C. A. Miller N. J. Papaga G. (1997). Antioxidant properties of phenolic compounds. 2 152–159. 10.1016/S1360-1385(97)01018-2

[B39] Rizzini L. Favory J. J. Cloix C. Faggionato D. O’Hara A. Kaiserli E. (2011). Perception of UV-B by the *Arabidopsis* UVR8 protein. 332 103–106. 10.1126/science.1200660 21454788

[B40] Rubery P. H. Sheldrake A. R. (1974). Carrier-mediated auxin transport. 118 101–121. 10.1007/BF00388387 24442257

[B41] Ruppel N. J. Hangarter R. P. Kiss J. Z. (2001). Red-light-induced positive phototropism in *Arabidopsis* roots. 212 424–430. 10.1007/s004250000410 11289607

[B42] Sabatini S. Beis D. Wolkenfelt H. Murfett J. Guilfoyle T. Malamy J. (1999). An auxin-dependent distal organizer of pattern and polarity in the *Arabidopsis* root. 99 463–472. 10.1016/S0092-8674(00)81535-4 10589675

[B43] Santelia D. Henrichs S. Vincenzetti V. Sauer M. Bigler L. Klein M. (2008). Flavonoids redirect PIN-mediated polar auxin fluxes during root gravitropic responses. 283 31218–31226. 10.1074/jbc.M710122200 18718912 PMC2662185

[B44] Silva-Navas J. Moreno-Risueño M. A. Manzano C. Téllez-Robledo B. Navarro-Neila S. Carrasco V. (2016). Flavonols mediate root phototropism and growth through regulation of proliferation-to-differentiation transition. 28 1372–1387. 10.1105/tpc.15.00857 26628743 PMC4944400

[B45] Solomon K. R. (2008). Effects of ozone depletion and UV-B radiation on humans and the environment. 46 185–202. 10.3137/ao.460109

[B46] Sun F. Zhang W. Hu H. Li B. Wang Y. Zhao Y. (2008). Salt modulates gravity signaling pathway to regulate growth direction of primary roots in *Arabidopsis*. 146 178–188. 10.1104/pp.107.109413 18024552 PMC2230569

[B47] Stenlid G. (1976). Effects of flavonoids on the polar transport of auxins. 38 262–266. 10.1111/j.1399-3054.1976.tb04001.x

[B48] Swarup R. Kargul J. Marchant A. Zadik D. Rahman A. Mills R. (2004). Structure-function analysis of the presumptive Arabidopsis auxin permease AUX1. 16 3069–3083. 10.1105/tpc.104.024737 15486104 PMC527199

[B49] Swarup R. Kramer E. M. Perry P. Knox K. Leyser H. M. Haseloff J. (2005). Root gravitropism requires lateral root cap and epidermal cells for transport and response to a mobile auxin signal. 7 1057–1065. 10.1038/ncb1316 16244669

[B50] Tsukagoshi H. Busch W. Benfey P. N. (2010). Transcriptional regulation of ROS controls transition from proliferation to differentiation in the root. 143 606–616. 10.1016/j.cell.2010.10.020 21074051

[B51] Ulmasov T. Murfett J. Hagen G. Guilfoyle T. J. (1997). Aux/IAA proteins repress expression of reporter genes containing natural and highly active synthetic auxin response elements. 9 1963–1971. 10.1105/tpc.9.11.1963 9401121 PMC157050

[B52] Vandenbussche F. Tilbrook K. Fierro A. C. Marchal K. Poelman D. Van Der Straeten D. (2014). Photoreceptor-mediated bending towards UV-B in *Arabidopsis*. 7 1041–1052. 10.1093/mp/ssu039 24711292

[B53] Vanhaelewyn L. Prinsen E. Van Der Straeten D. Vandenbussche F. (2016). Hormone-controlled UV-B responses in plants. 67 4469–4482. 10.1093/jxb/erw261 27401912

[B54] Winkel-Shirley B. (2002). Biosynthesis of flavonoids and effects of stress. 5 218–223. 10.1016/S1369-5266(02)00256-X 11960739

[B55] Wolverton C. Ishikawa H. Evans M. L. (2002). The kinetics of root gravitropism: dual motors and sensors. 21 102–112 10.1007/s003440010053 12024226

[B56] Yan S. Zhang T. Dong S. McLamore E. S. Wang N. Shan X. (2016). MeJA affects root growth by modulation of transmembrane auxin flux in the transition zone. 35 256–265. 10.1007/s00344-015-9530-9

[B57] Yin R. Han K. Heller W. Schaffner A. R. (2014). Kaempferol 3-*O*-rhamnoside-7-*O*-rhamnoside is an endogenous flavonol inhibitor of polar auxin transport in *Arabidopsis* shoots. 201 466–475. 10.1111/nph.12558 24251900 PMC4260840

[B58] Yokawa K. Kagenishi T. Baluška F. (2016). UV-B induced generation of reactive oxygen species promotes formation of BFA-induced compartments in cells of *Arabidopsis* root apices. 6:1162. 10.3389/fpls.2015.01162 26793199 PMC4710705

[B59] Žádníková P. Petrášek J. Marhavý P. Raz V. Vandenbussche F. Ding Z. (2010). Role of PIN-mediated auxin efflux in apical hook development of *Arabidopsis thaliana*. 137 607–617. 10.1242/dev.041277 20110326

[B60] Zhang K. X. Xu H. H. Yuan T. T. Zhang L. Lu Y. T. (2013). Blue-light-induced PIN3 polarization for root negative phototropic response in *Arabidopsis*. 76 308–321. 10.1111/tpj.12298 23888933

[B61] Zhao Y. D. Christensen S. K. Fankhauser C. Cashman J. R. Cohen J. D. Weigel D. (2001). A role for flavin monooxygenase-like enzymes in auxin biosynthesis. 291 306–309. 10.1126/science.291.5502.306 11209081

