# Supplementary material for: UV-B Radiation Induces Root Bending Through the Flavonoid-Mediated Auxin Pathway in Arabidopsis
Source: Front Plant Sci. 2018 May 17;9:618. doi: 10.3389/fpls.2018.00618 (PMC5966577; doi:10.3389/fpls.2018.00618)
Supplement: Supplementary file 2 [file Presentation_1.PDF]

Supporting Information

Table S1. List of the primers for qRT-PCR analysis of the genes.

Gene	Primers
4CL3	5’AAAGGCTTTCAGGTCCCTCC3’<br>5’AGAACACCACCTGTTTGGCA3’
CHI	5’TAGTCACCGGTGCGTTTGAG3’<br>5’AGAGGATCGATGAACCGGGA3’
CHI-L1	5’ACCACCAGTCCCTGGCTTAT3’<br>5’GTCTGTGATCCCTTGGCCC3’
F3H	5’TCGCTCGTGACTTCTTTGCT3’<br>5’CCTCTCCCTGGAGGTGACTA3’
FLS1	5’CCACCTGAATACAGGGAGGT3’<br>5’TGAGCCGGTACACCTAAAGC3’
F3GlcT(UGT78D2)	5’TCCGGAGAGAAATCGCGAAG3’<br>5’CGCTCACCTACTTCTTTGACAC3’
F7GlcT(UGT73C6)	5’TGGCTCAAGGCCACATGATT3’<br>5’TCTGCTCCATCGTGGTAAGC3’
F7RhaT(UGT89C1)	5’GGCGTTGGAGAAAAGCAGTG3’<br>5’TGGGGCCCATCCTCTTATCA3’
SCPL10	5’TGTCAAAGGAGGTGGACACAC3’<br>5’AAAGAATTGCGCGTAGCGTG3’
PAL2	5’TCATTAGATTTTTGAACGCCGGA3’<br>5’CGGAGAGGTAGTGACGGAGA3’
F3'H(CYP75B1)	5’TACGGACACCGATGGAGACT3’<br>5’GCGTTCCAACCTCTTCCTGT3’
AtYUC2	5’GGTGACACGGATCGGTTAGGGT3’<br>5’TGCCGAATAATGCATTACCCGT3’
AtYUC3	5’CTTGAGATTGATTCCGTTATTC3<br>5’GGAGAAGAAGTCGTTGTC3’
AtYUC9	5’ATCTTGCTAACCACAATG3’<br>5’CCACTTCATCATCATCAC3
AtSUR1	5’GACCACCAAGGTGTTACAATCC3’<br>5’ATTATTGTGGCAGGGTCAGG3’
AtAAO3	5’GGAGTCAGCGAGGTGGAAGT3’<br>5’TGCTCCTTCGGTCTGTCCTAA3’
AtASA1	5’ATGTCTTCCTCTATGAACGTAGC3’<br>5’ACAGCGGTAAATTGGTATAAGG3’
AtTAA1	5’CTCCAAGATCACAGGCCACGCTGGG3’<br>5’GACTCCTTAGACACACCAATCGAGTTC3’
AtPAT1	5’ATGGTTATTGCGGTGGCGAC3’

	5’ATCGTCGCCGACTCAATGTC3’
AtCYP79B2	5’CACGATGATGCTCGCGAGACT3’<br>5’TCACTTCACCGTCGGGTAGAGA3’
AtHPS7/AQC1	5’GGAGCGCACACAATCTCTCT3’<br>5’GTGCCTCGATTCCAAACCCT3’

Table S2. List of Differentially Expressed Genes in roots.

Please see the EXCEL file **Supplemental Table S2.**

Table S3. Differential expression genes involved in the flavonoid biosynthesis by RNA-seq analysis. ck, before UV-B radiation control.

**Gene**	**0.5 h/ck**		**2 h/ck**		**Annotation**
	**log2FC**	**FDR**	**log2FC**	**FDR**	
**Flavonoid biosynthesis**					
AT1G65060	2.028909062	3.17E-09	1.987584159	3.12E-06	*4CL3*
AT3G55120	1.398721749	0.0002427	-	-	*CHI*
AT5G05270	1.458694923	9.63E-05	1.586105887	0.0008203	*CHI-L1*
AT3G51240	2.092014527	2.39E-10	2.013333854	2.13E-06	*F3H*
AT5G08640	1.663145351	2.81E-06	1.481675326	0.0041158	*FLS1*
AT5G17050	2.61981105	4.44E-16	1.682227088	0.0001935	*F3GlcT(UGT78D2)*
AT2G36790	1.810261212	0.006434	5.044925006	0	*F7GlcT(UGT73C6)*
AT1G06000	1.547685997	2.13E-05	-	-	*F7RhaT(UGT89C1)*
AT2G23000	2.115949338	1.27E-05	-	-	*SCPL10*
AT3G53260	-	-	1.505608279	0.0072008	*PAL2*
AT5G07990	-	-	1.576975258	0.0011331	*F3'H(CYP75B1)*

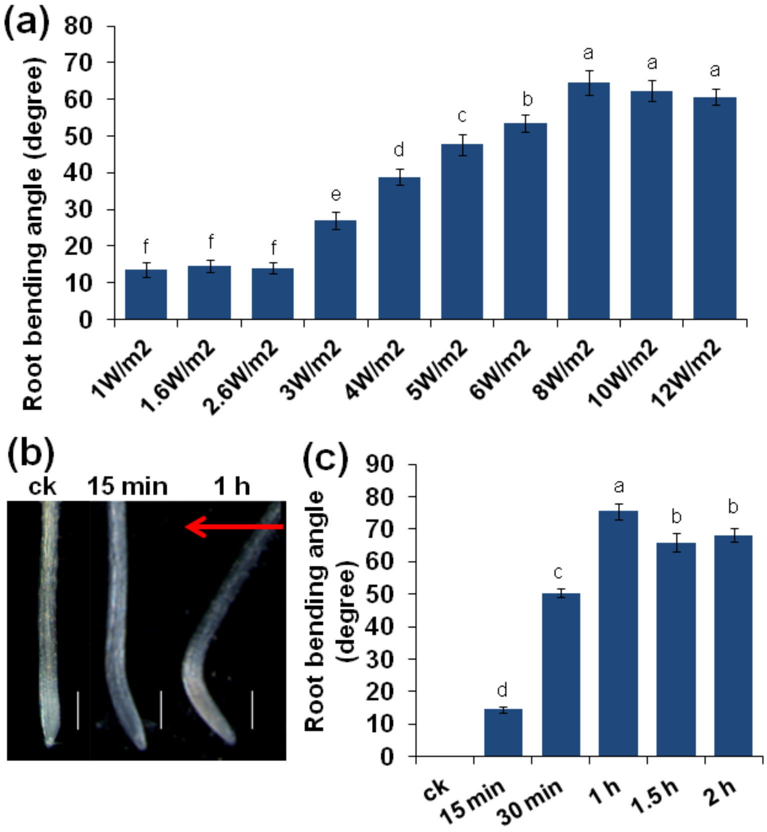

Fig. S1 UV-B radiation induces root bending. (a) Five-day-old *Arabidopsis* seedlings grown in 1/2 MS medium were irradiated with 1-12 W m^{-2} of UV-B radiation for 15 min and then transferred to normal growth conditions, the angles of root bending was measured after 4 h of treatment. (b) Images of 5-d-old *Arabidopsis* seedlings grown on vertical plates, irradiated with 1.6 W m^{-2} UV-B radiation for 15 min and 1 h and then transferred to normal growth conditions for 4 h. Bars, 0.5 cm. (c) Five-day-old *Arabidopsis* seedlings grown in 1/2 MS medium were irradiated with 1.6 W m^{-2} UV-B radiation for 15 min-2 h and then transferred to normal growth conditions for 4 h; the angles of root bending was measured. ck, before UV-B radiation control. The error bars represent the ± SE, and different letters indicate significantly different values ($P<0.01$ by Tukey's test).

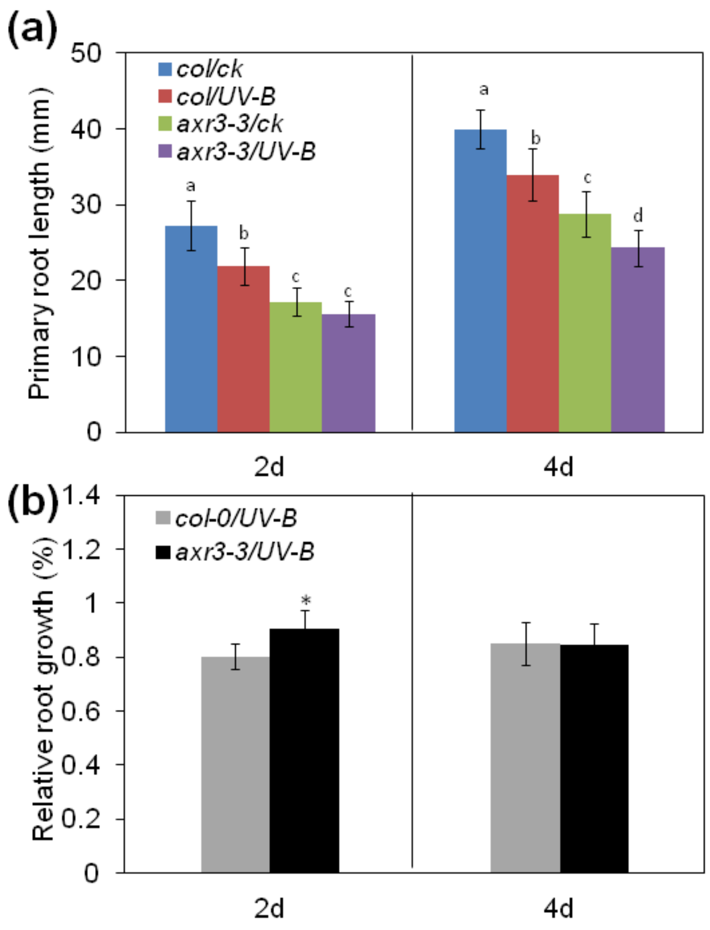

Fig. S2 Five-day-old *Arabidopsis col-0* and *axr3-3* seedlings grown in 1/2 MS medium were irradiated with 1.6 W m^{-2} UV-B radiation for 1 h and then transferred to normal growth conditions for 2 and 4 d. (a) The PR length and (b) the relative root growth of *col-0* and *axr3-3* seedlings treated with UV-B compared with untreated seedlings. ck, non-irradiated control. The error bars represent the ± SE. Different letters indicate significantly different values (P < 0.01 by Tukey's test). Asterisks (*) indicate significant differences with respect to the corresponding *col-0* seedlings (P < 0.01 by Tukey's test).

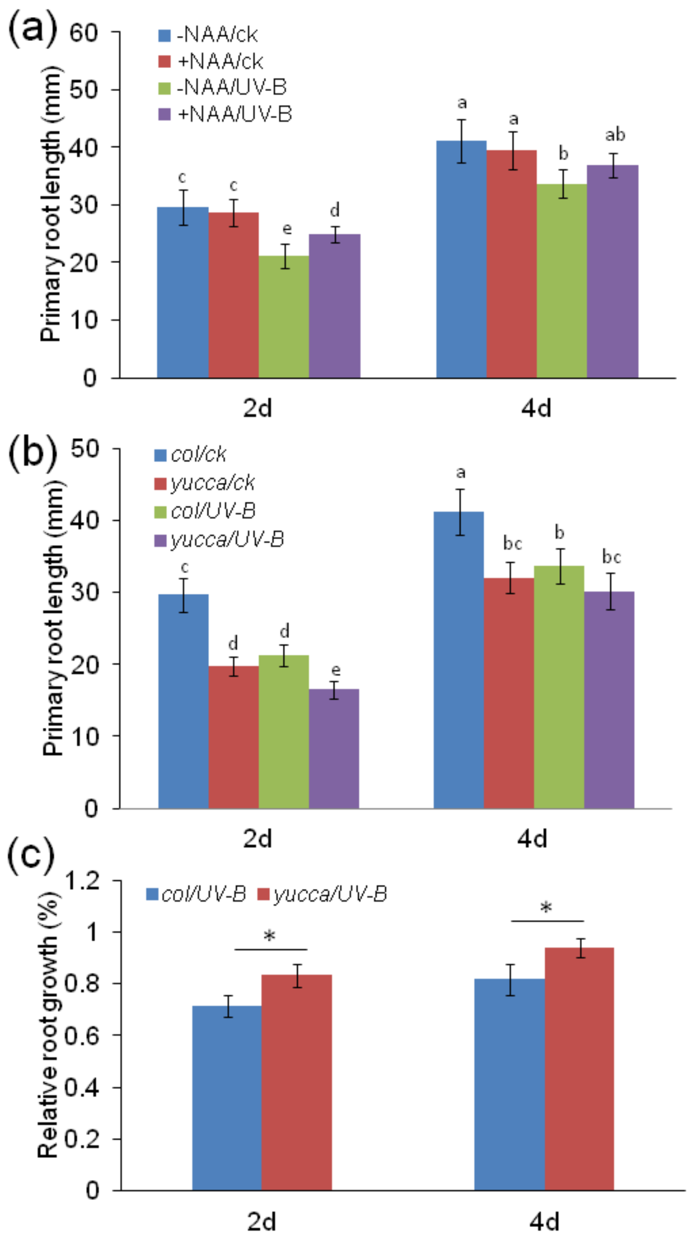

Fig. S3 (a) Five-day-old *Arabidopsis col-0* seedlings grown in 1/2 MS medium with or without 10 nM NAA were irradiated with 1.6 W m^{-2} UV-B radiation for 1 h and then transferred to normal growth conditions for 2 and 4 d; the PR length was measured. (b, c) Five-day-old *Arabidopsis col-0* and *yucca* seedlings grown in 1/2 MS medium were irradiated with 1.6 W m^{-2} UV-B radiation for 1 h and then transferred to normal growth conditions for 2 and 4 d. (b) The PR length and (c) the relative root growth of *col-0* and *yucca* seedlings treated with UV-B compared with untreated seedlings. ck, non-irradiated control. The error bars represent the ± SE. Different letters indicate significantly different values ($P < 0.01$ by Tukey's test). Asterisks (*) indicate significant differences with respect to the corresponding *col-0* seedlings ($P < 0.01$ by Tukey's test).

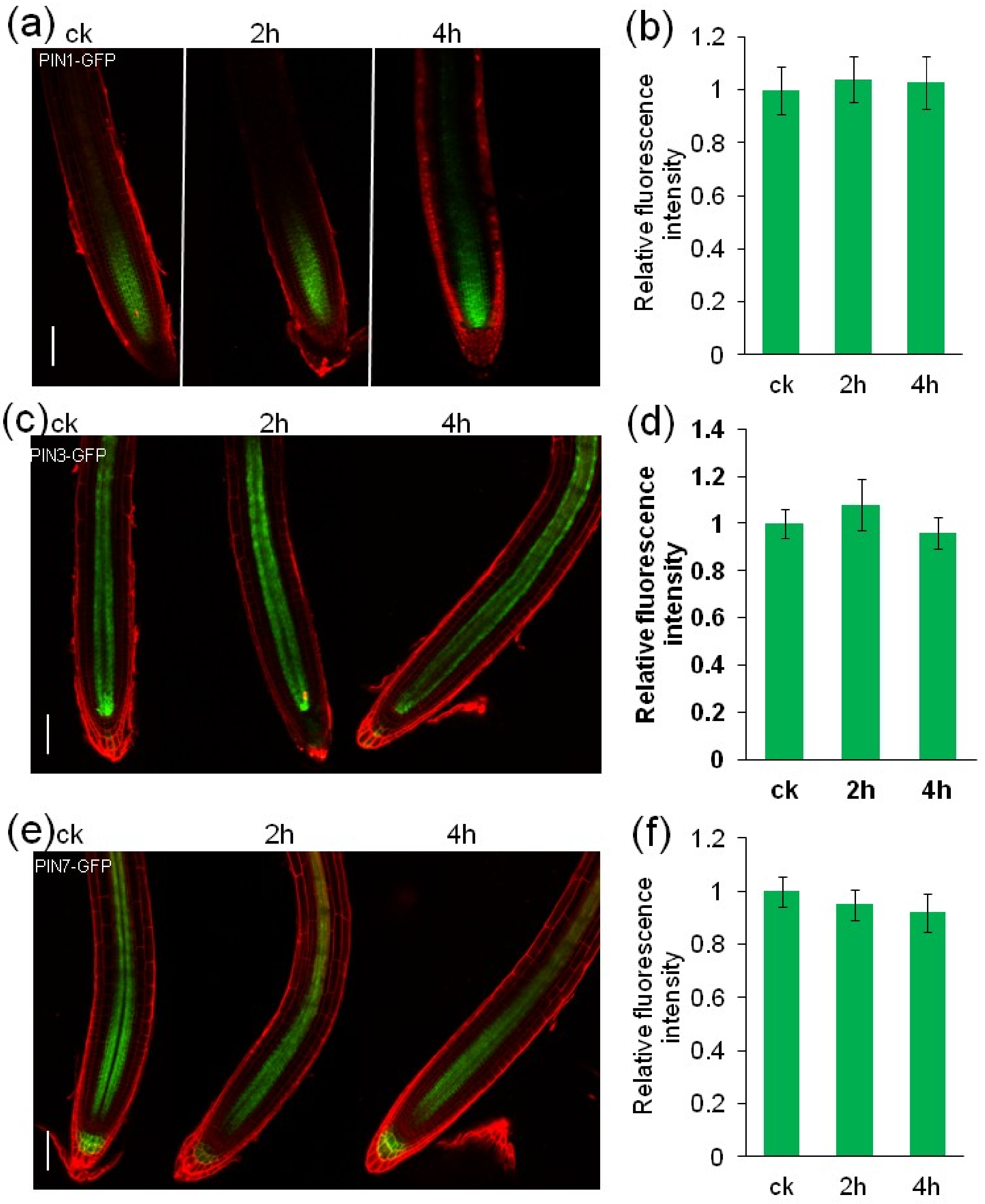

Fig. S4 GFP fluorescence in the roots of 5-d-old *PIN1::GFP* (a), *PIN3::GFP* (c) and *PIN7::GFP* (e) seedlings exposed to 1.6 W m^{-2} UV-B radiation for 1 h and then transferred to normal growth conditions for 2 and 4 h and the quantification of *PIN1::GFP* (b), *PIN3::GFP* (d), and *PIN7::GFP* (f) fluorescence intensities in plants treated as in (a, b, and c, respectively). Bars, 60 μm. The error bars represent the SE. ck, before UV-B radiation control.

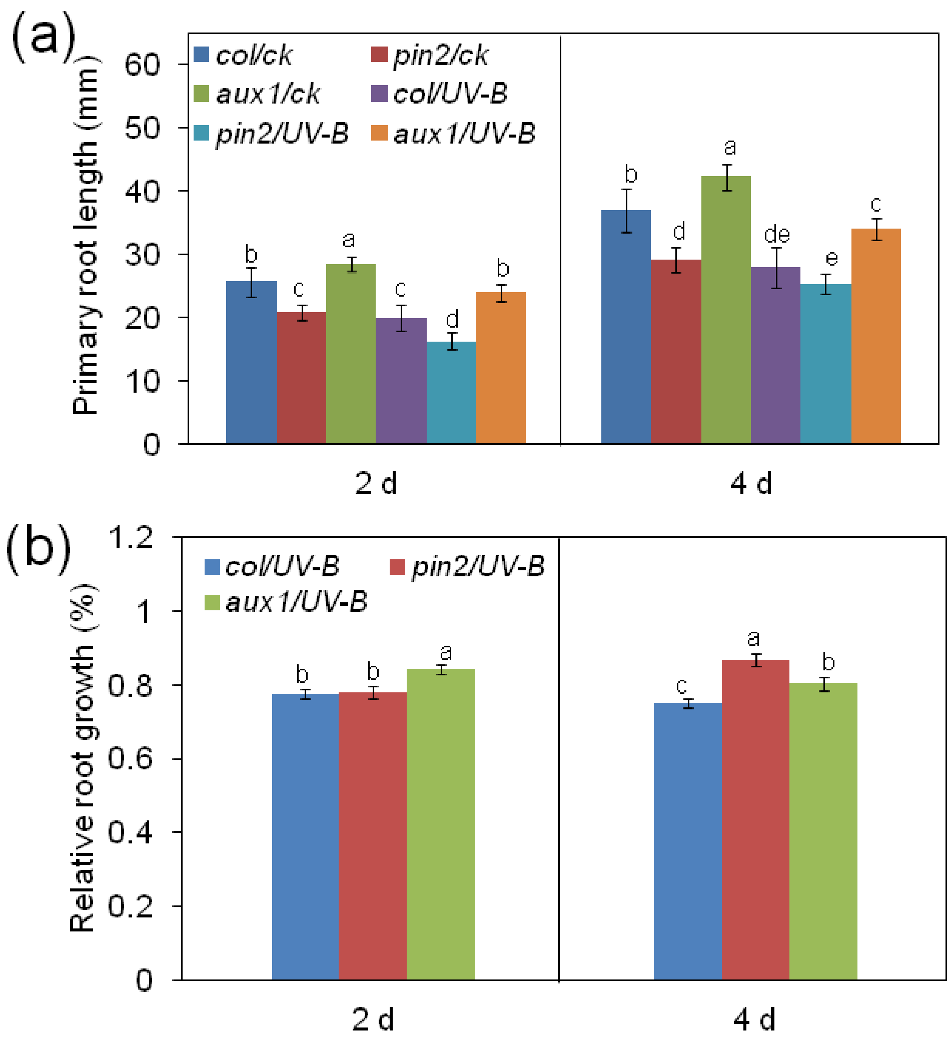

Fig. S5 Five-day-old *Arabidopsis col-0*, *pin2*, and *aux1-7* seedlings grown in 1/2 MS medium were irradiated with 1.6 W m^{-2} UV-B radiation for 1 h and then transferred to normal growth conditions for 2 and 4 d. (a) The PR length and (b) the relative root growth of *col-0*, *pin2*, and *aux1-7* seedlings treated with UV-B compared with untreated seedlings. ck, non-irradiated control. The error bars represent the ± SE. Different letters indicate significantly different values ($P < 0.01$ by Tukey's test).

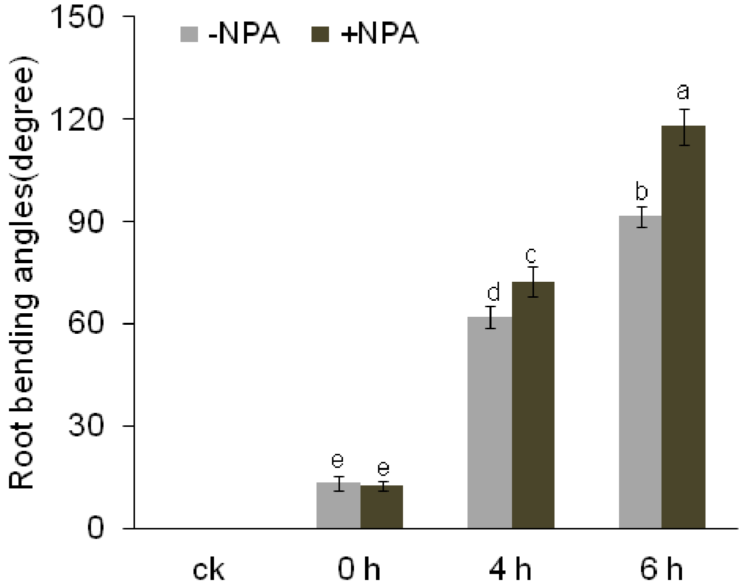

Fig. S6 The angle of root bending of the wild-type *col-0* seedlings irradiated with 1.6 W m^{-2} UV-B radiation for 1 h and then transferred to normal growth conditions plus 0 μM NPA or 1 μM NPA for 0-6 h. ck, before UV-B radiation control. The error bars represent the ± SE. Different letters indicate significantly different values ($P < 0.01$ by Tukey's test).

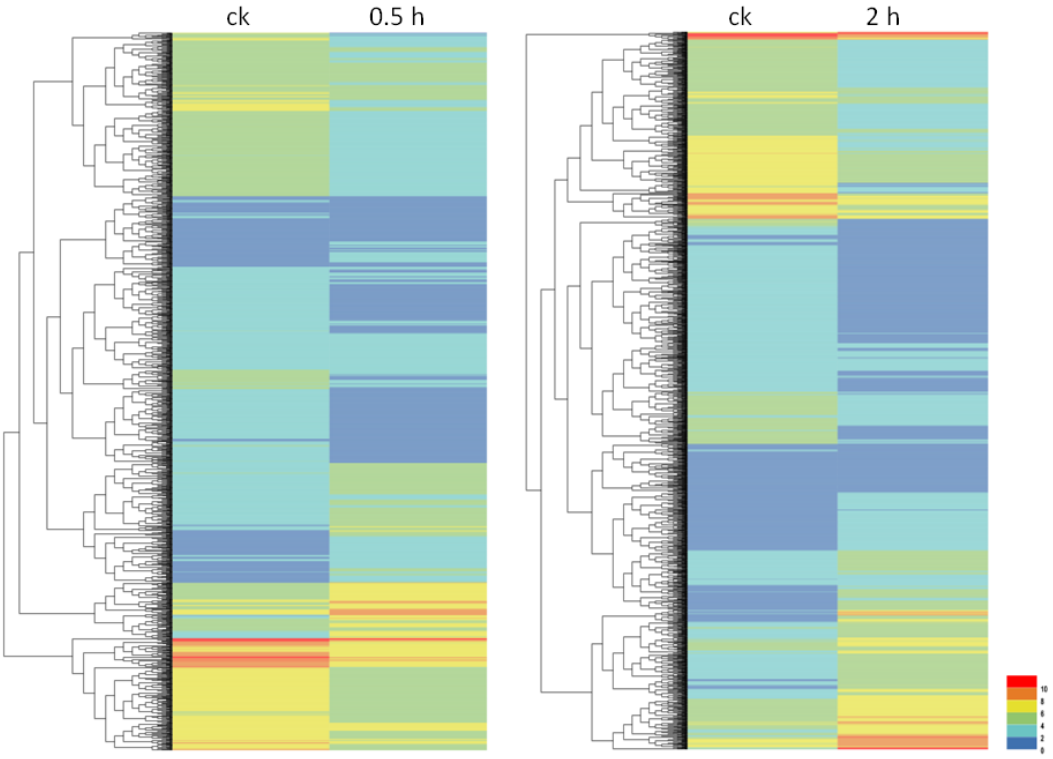

Fig. S7 Hierarchical clustering analysis of the differentially expressed genes in *Arabidopsis* roots after 0.5 h or 2 h of UV-B treatment. ck, before UV-B radiation control.

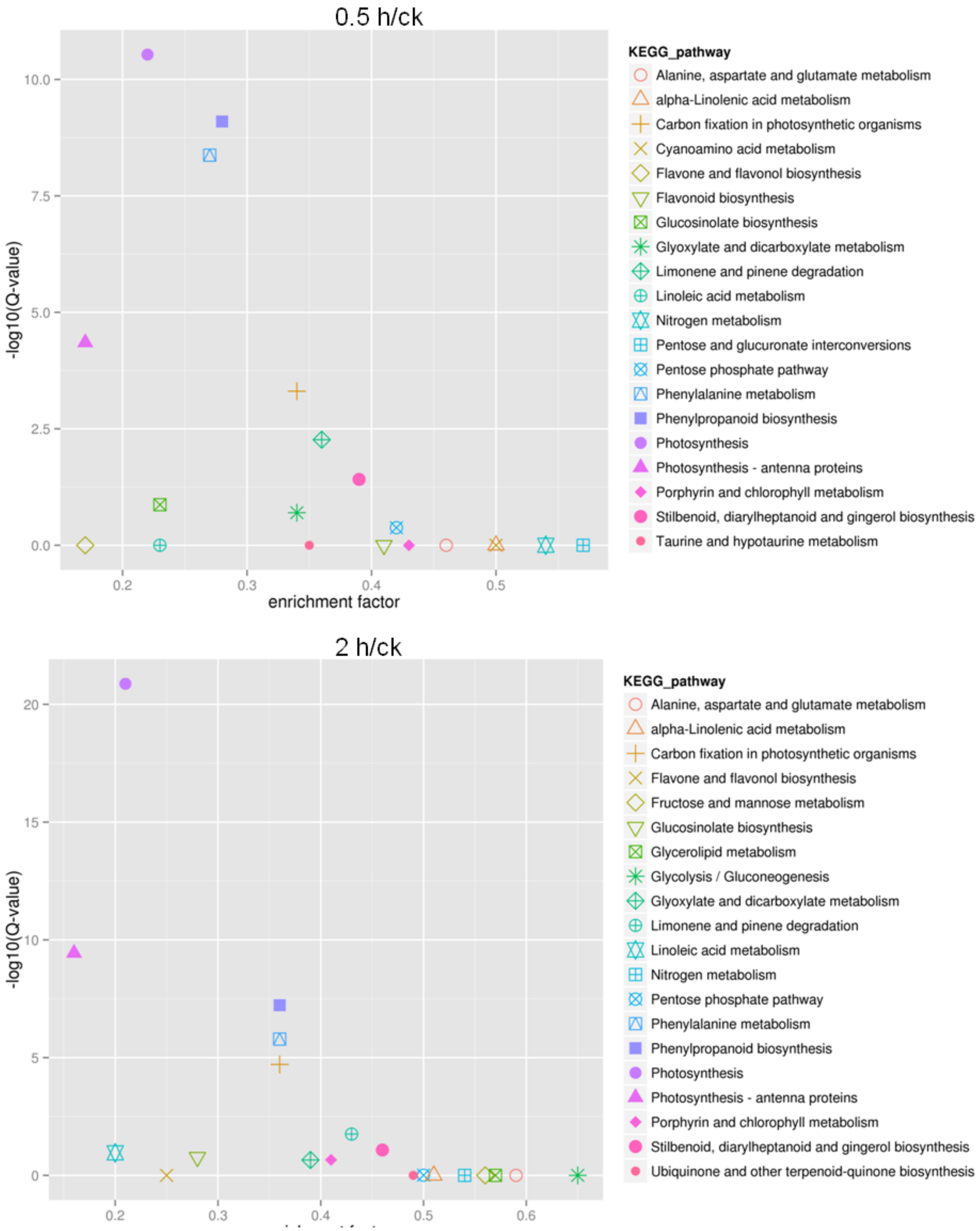

Fig. S8 KEGG pathway enrichment analysis of the differentially expressed genes in *Arabidopsis* roots after 0.5 h or 2 h of UV-B treatment. ck, before UV-B radiation control.

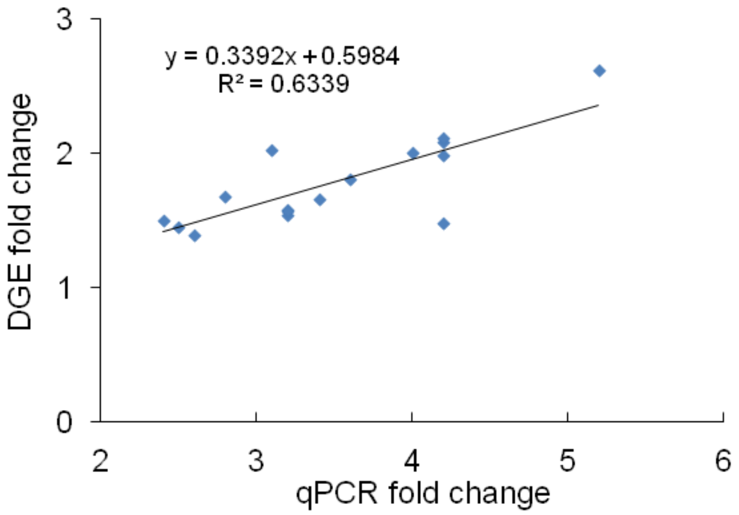

Fig. S9 Reliability analysis of the RNA-seq data based on comparison with the qRT-PCR results.

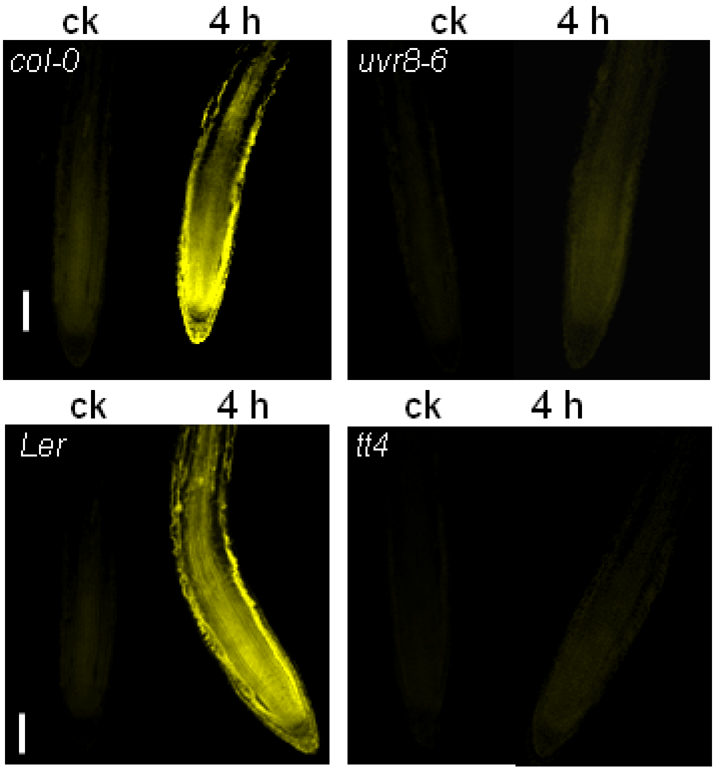

Fig. S10 DPBA fluorescence in the roots of 5-d-old Col-0, *uvr8-6*, *Ler*, and *tt4* seedlings exposed to 1.6 W m^{-2} UV-B radiation for 1 h and then transferred to normal growth conditions for 4 h. Bars, 100 μm.

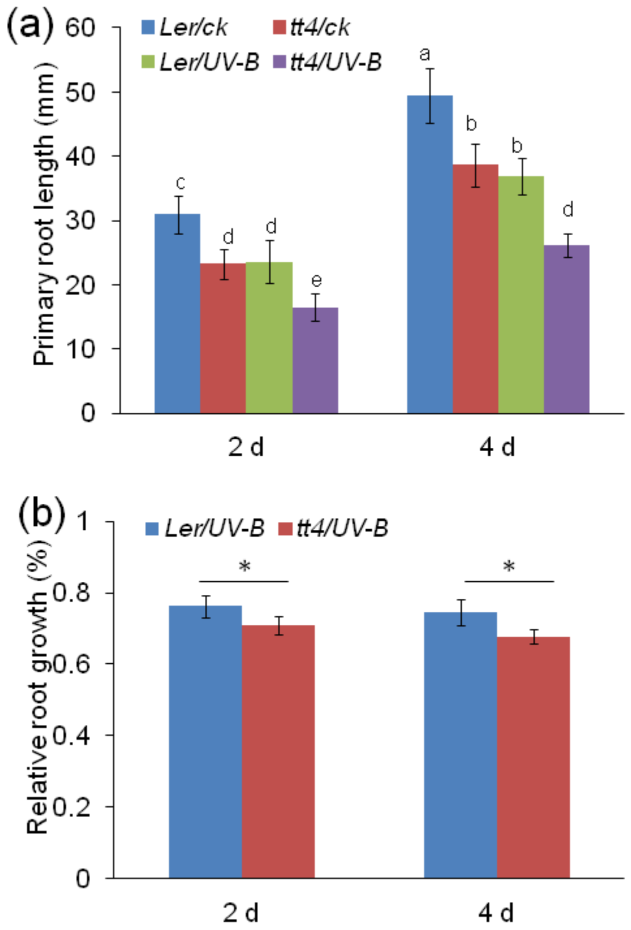

Fig. S11 Five-day-old *Arabidopsis Ler* and *tt4-1* seedlings grown in 1/2 MS medium were irradiated with 1.6 W m^{-2} UV-B radiation for 1 h and then transferred to normal growth conditions for 2 and 4 d. (a) The PR length and (b) the relative root growth of *Ler* and *tt4-1* seedlings treated with UV-B compared with untreated seedlings. ck, non-irradiated control. The error bars represent the ± SE. Different letters indicate significantly different values ($P < 0.01$ by Tukey's test).

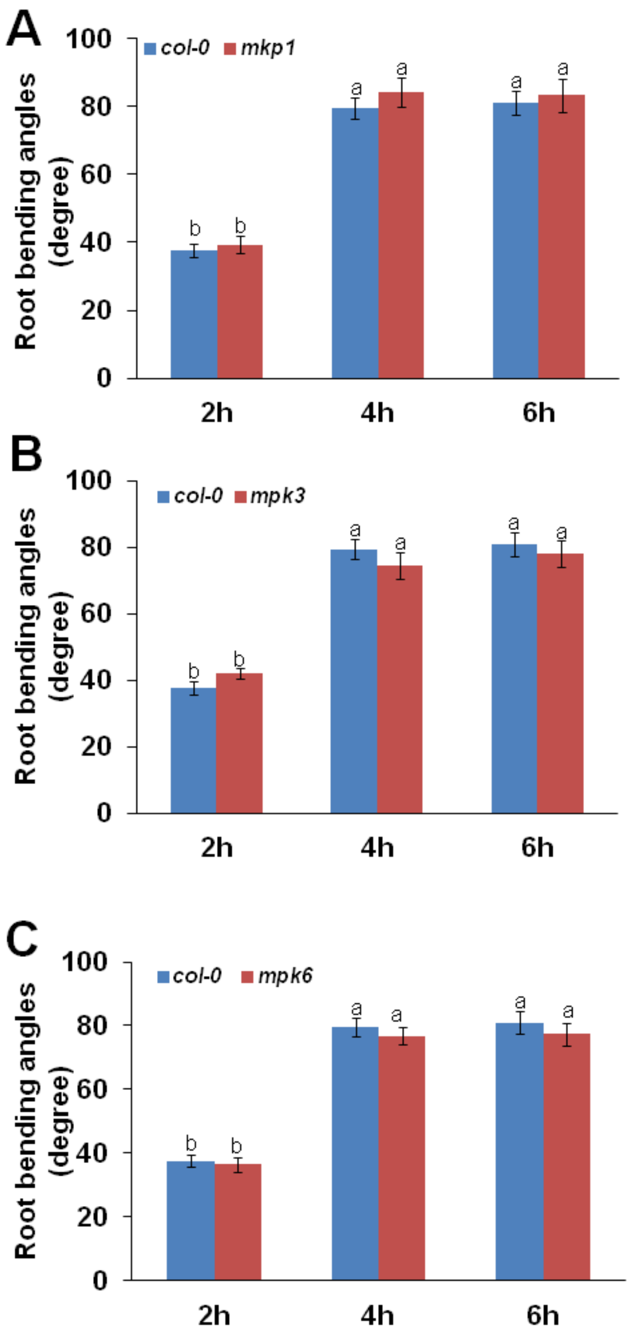

Fig. S12 Angles of root bending of wild-type *col-0*, *mkp1*, *mpk3*, and *mpk6* seedlings irradiated with 1.6 W m^{-2} UV-B radiation for 1 h and then transferred to normal growth conditions for 2-6 h. The error bars represent the ± SE, and different letters indicate significantly different values ($P < 0.01$ by Tukey's test).

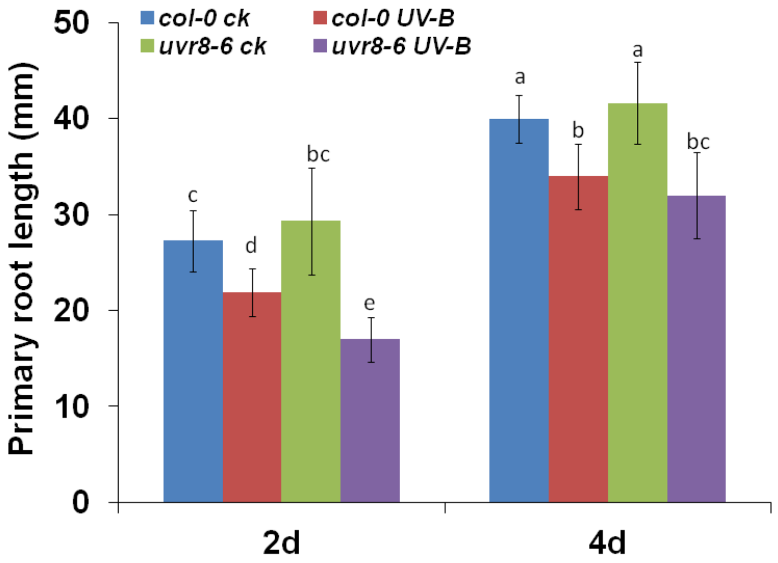

Fig. S13 Five-day-old *Arabidopsis col-0* and *uvr8* seedlings grown in 1/2 MS medium were irradiated with 1.6 W m^{-2} UV-B radiation for 1 h and then transferred to normal growth conditions for 2 and 4 d; the PR length was measured. ck, non-irradiated control. The error bars represent the ± SE, and different letters indicate significantly different values (P<0.01 by Tukey's test).

Supplementary Materials and methods

Digital Transcriptomics and Screening of Differentially Expressed Genes

Five-day-old *Arabidopsis* seedlings were irradiated with UV-B (2.6 kJ m^{-2}) for 3 min in the presence of simultaneous natural white light radiation and then transferred to normal growth conditions for 0.5 and 2 h. The RNAs were extracted from the roots of the control (0 h) and treated (0.5 h and 2 h) seedlings using RNAiso Plus (TaKaRa) according to the manufacturer's instructions. The RNA quality and integrity were checked before the cDNA was synthesized using the PrimeScript^{TM} RT Reagent Kit with gDNA Eraser (TaKaRa). Oligo (dT) magnetic bead adsorption was used to purify the mRNA, and the mRNA was randomly fragmented using fragmentation buffer. The 6-bp-random hexamers were used as a primer to synthesize the first and second strand cDNA. AMPure XP beads were used to purify the cDNA. After end repair and adding A tail, the purified double-strand cDNA was linked with the sequencing adaptor and then subjected to fragment size selection using AMPure XP beads. The cDNA library was obtained by PCR amplification. The concentration of the cDNA library was analyzed using Qubit 2.0 and Agilent 2100 and was quantified using Q-PCR. High-throughput sequencing was performed using Hiseq 2500, and the read length of the sequencing was SE50. The raw sequences were transformed into clean tags after certain steps of data processing were performed, including the removal of the adaptor sequence, empty reads, and low-quality tags; finally, clean tags were generated. Sequences from Arabidopsis (TAIR 10) were used to obtain the reference gene sequence. All of the clean tags were mapped to the reference sequences. The clean tags that mapped to the reference sequences of multiple genes were filtered.

The differentially expressed genes were screened using EBSeq (Leng et al., 2013). The P value corresponds to the differential gene expression test. The FDR (False Discovery Rate) is a method to determine the threshold of the P value in multiple tests and analyses by manipulating the FDR value. We used the FDR ≤ 0.01 and the absolute value of log2Ratio > 1 as the threshold to judge the significance of the constitutive and induced gene expression differences.

High-throughput sequencing was performed with the assistance of Biomarker Corporation (Beijing, China). All sequence data for this study were archived at the National Center for Biotechnology Information's Short Read Archive (SRA) under accession no SRP094914.

Supplementary References

Leng N, Dawson JA, Thomson JA, et al. 2013. EBSeq: an empirical Bayes hierarchical model for inference in RNA-seq experiments. *Bioinformatics* **29:** 1035-1043.
